# Autophagy stimulation as a promising approach in treatment of neurodegenerative diseases

**DOI:** 10.1007/s11011-018-0214-6

**Published:** 2018-03-14

**Authors:** Karolina Pierzynowska, Lidia Gaffke, Zuzanna Cyske, Michał Puchalski, Estera Rintz, Michał Bartkowski, Marta Osiadły, Michał Pierzynowski, Jagoda Mantej, Ewa Piotrowska, Grzegorz Węgrzyn

**Affiliations:** Department of Molecular Biology, Faculty of Biology, University of Gdańsk, Wita Stwosza 59, 80-308 Gdańsk, Poland

**Keywords:** Autophagy stimulation, Lysosomes, Therapeutic strategies, Neurodegenerative diseases

## Abstract

Autophagy is a process of degradation of macromolecules in the cytoplasm, particularly proteins of a long half-life, as well as whole organelles, in eukaryotic cells. Lysosomes play crucial roles during this degradation. Autophagy is a phylogenetically old, and evolutionarily conserved phenomenon which occurs in all eukaryotic cells. It can be found in yeast *Saccharomyces cerevisiae,* insect *Drosophila melanogaster*, and mammals, including humans. Its high importance for cell physiology has been recognized, and in fact, dysfunctions causing impaired autophagy are associated with many severe disorders, including cancer and metabolic brain diseases. The types and molecular mechanisms of autophagy have been reviewed recently by others, and in this paper they will be summarized only briefly. Regulatory networks controlling the autophagy process are usually described as negative regulations. In contrast, here, we focus on different ways by which autophagy can be stimulated. In fact, activation of this process by different factors or processes can be considered as a therapeutic strategy in metabolic neurodegenerative diseases. These aspects are reviewed and discussed in this article.

## The autophagy process

In healthy non-stressed cells, synthesis and degradation of macromolecules (proteins, nucleic acids, lipids, polysaccharides) occur generally at roughly constant levels. Proteins, as major functional macromolecules, are crucial to maintain cellular homeostasis. Therefore, protein synthesis and degradation must be balanced in cells. Any disturbance in this balance may lead to severe dysfunctions of the cell, a group of cells, tissues, organs and the whole organism, as a result of a complex network between various biological processes.

One of two major systems for protein degradation in eukaryotic cells, beside the proteasomal pathway, is the process of lysosome-mediated degradation, called autophagy. This process is phylogenetically old, evolutionarily conserved phenomenon which occurs in all eukaryotic cells. It can be found in yeast *Saccharomyces cerevisiae,* insect *Drosophila melanogaster*, and mammals, including humans. (Ricci and Zong 2006). It is employed mainly to degrade macromolecules in the cytoplasm, particularly proteins of a long half-life, as well as whole organelles, and lysosomes play crucial roles during this degradation (Meijer and Codogno 2004). Randomly selected part of the cytoplasm, together with its compounds, can undergo the digestion, and this kind of the process is called non-selective autophagy. It is employed to maintain the equilibrium in the amount and size of particular components of the cytoplasm. The selective autophagy occurs when special organelles or structures are subjected to degradation, for example mitochondria (the process is called mitophagy), endoplasmic reticulum (reticulophagy), or ribosomes (ribophagy) (Liang and Jung 2010).

The autophagy occurs under physiological conditions (called the basic autophagy), and it is involved in the maintenance of cellular homeostasis. However, it can be stimulated in response to various stress conditions (called the induced autophagy), including oxidative stress (appearance of reactive oxygen species), unfolded proteins, viral infection or starvation. The latter process has a role in the adaptation to new, unfavorable conditions, when the cell is deprived of compounds for the synthesis of new molecules, crucial for survival under stress conditions (Ricci and Zong 2006).

On the basis of the type of delivery of the substrate to lysosomes, three major forms of autophagy have been distinguished: (i) microautophagy, (ii) macroautophagy, and (iii) chaperone-dependent autophagy (Cuervo 2004). Microautophagy is the process devoted to degradation of small organelles and compounds suspended in the cytoplasm (Sakai et al. 1998). In this process, a fragment of cytoplasm is sequestered directly by a lysosome due to invagination of lysosomal membrane (Mijaljica et al. 2011). The chaperone-dependent autophagy involves binding of individual proteins or peptides by chaperones from the Hsp70 family and formation of the chaperone-substrate complexes. Such complexes are transported into lysosomes after being recognized by the receptors Lamp2a (lysosome-associated membrane protein type 2a) and are degraded inside these organelles (Kaushik et al. 2011). Unlike the two mechanisms described above, in which the only organelle necessary to conduct the autophagy process is lysosome, in the case of macroautophagy, a fusion between lysosome and autophagosome is required to degrade selected cellular structures. Macroautophagy is the most common type of autophagy. In the initial step of this process, a fragment of cytoplasm, together with proteins and/or organelles, is engulfed by the phagophore, a double membrane structure. Capturing of the cytoplasm fragment results in formation of autophagosome, to which early and late endosomes can fuse, providing factors required to further fusion with lysosome, as well as factors lowering pH to form acid environment, necessary for activities of lysosomal hydrolases. So prepared autophagosome fuses to lysosome, which leads to formation of the autophagolysosome. Internal membrane of the autophagosome is then degraded, together with organelles and macromolecules included in it. Degradation products (amino acids, nucleotides, simple carbohydrates, fatty acids) are then released to the cytoplasm and can be used in various cellular processes (Dong and Czaja 2011; Ricci and Zong 2006; Cuervo 2004).

## The role of autophagy in the cell

In eukaryotic cells, the autophagy process has multiple functions. In the normally functioning cell, this process occurs at a constant level, and it is called basic or constitutive autophagy. It is responsible for maintenance of cellular homeostasis by removal of damaged or unnecessary organelles or regulation of the size of endoplasmic reticulum (Qu et al. 2007). Moreover, it facilitates the balance between synthesis and degradation of macromolecules. Basic autophagy is involved also in various physiological processes, like neurolamine synthesis in dopaminergic neurons, surfactant biogenesis in pneumocytes, or erythrocyte maturation (Kim 2005). It also has a role in yeast sporulation, regression of the mammary gland in cattle (Zarzynska and Motyl 2008), and nymph development in *Drosophila melanogaster* (Yang et al. 2005). Autophagy is also necessary for implantation of the mouse embryo into uterus, and its first cellular divisions (Tsukamoto et al. 2008). Mutations in the *Atg*5 gene, coding for a protein responsible for autophagy initiation, result in death of mice shortly after they were born (Kuma et al. 2004). Inactivation of the gene coding for Beclin 1 caused a decrease in life span of *Caenorhabditis elegans* (Meléndez et al. 2003). In contrast, overexpression of the *Atg8* gene, which product is involved in building of the autophagosome membrane, resulted in longer life span of *D. melanogaster* by about 50% (Vellai 2009). It is assumed that this effect depends on one of selective autophagy processes, the mitophagy, in which damaged mitochondria are removed from the cell. This, in turn, restricts the appearance of reactive oxygen species in the cell, and/or facilitates repair of DNA and proteins (Shintani and Klionsky 2004).

The autophagy process is enhanced under conditions of the cellular stress. This process is called the induced autophagy. Among its inductors, there are starvation, a lack of growth factors, viral infection and DNA damage. Under such conditions, the autophagy facilitates adaptation of the cell to new environmental conditions, as it ensures the availability of compounds necessary for synthesis of macromolecules, required during the stress, through degradation of structures that are less important under such conditions. The autophagy is also employed to protect the infected cell from multiplication of viruses or bacteria (Klionsky 2005; Yang et al. 2005). However, long-term and intensive autophagy may lead to cell death, which is called programmed cell death (apoptosis) type II or autophagy-associated apoptosis. This kind of cell death proceeds through condensation of chromatin and degradation of cellular structures, including endoplasmic reticulum, Golgi apparatus, and ribosomes. Contrary to programmed cell death type I (classical apoptosis), the type II of this process is caspase-independent, and requires increased activities of lysosomal enzymes (Qu et al. 2007).

It is worth to note that both over-activity and halting of autophagy can be deleterious for the cell. Inhibition of this process for a longer time may lead to tumorigenesis due to disturbance in cell growth and genome instability (Liu et al. 2010). On the other hand, induction of autophagy in tumor cells can facilitate their survival under hypoxia, deficiency of nutrients, and during chemotherapy.

## Molecular mechanism of autophagy

Molecular mechanism of autophagy has been elucidated for the first time during studies on yeast *S. cerevisiae*. In fact, results of those studies have been recognized as a breakthrough in understanding of cellular processes, and the principal investigator, Yoshinori Ohsumi, has been awarded the Nobel Prize in 2016 (https://www.nobelprize.org/nobel_prizes/medicine/laureates/2016/press.html).

Genetic analyses of yeast cells led to identification of 32 genes, coding for proteins taking part in the autophagy process. Such genes are evolutionarily conserved, from yeast to mammals. Therefore, it was proposed to use a common nomenclature of the autophagy genes, consisting of the atg abbreviation (after AuTophaGy-related genes), followed by a consecutive number. Generally, the autophagy process depends on a cascade of interactions between Atg proteins (Kost et al. 2011).

The macroautophagy process (called „autophagy” further in the text) can be divided into 4 stages: (i) initiation, consisting of the synthesis of the isolating membrane, called phagophore, (ii) nucleation and elongation of the isolating membrane, which subsequently closes up, forming the autophagosome structure, (iii) fusion of the autophagosome with lysosomal membrane, which leads to formation of the autophagolysosome, (iv) degradation of the autophagolysosome content, together with its internal membrane, by lysosomal enzymes (Wong et al. 2011). Below, these stages are described in more detail.

### Initiation

Up to now, it is not clear what is the origin of the isolating membrane (phagophore), which is a prerequisite of the autophagosome. There are two hypotheses which may explain this process. The first hypothesis suggests that the membrane may be a part of endoplasmic reticulum or Golgi apparatus (Yorimitsu and Klionsky 2005). According to the second hypothesis, the phagophore is synthesized in the cytoplasm *de novo* (Yang et al. 2005). Most researchers working on the autophagy support the former hypothesis because the transmembrane protein Atg9 is localized in membranes of the late endosomes and in the trans vesicles of the Golgi apparatus. During starvation, Atg9 circulates between Golgi apparatus or late endosomes and newly formatted isolation membrane, providing compounds necessary for autophagosome creation (Yang et al. 2010). The Atg9 protein is present in the phagophore, but it could not be detected on the surface of already formed autophagosomes (Kost et al. 2011). This is caused by removal of this protein from the membrane by the Atg1 protein, which is a serine-threonine kinase, a component of the Atg1-Atg13-Atg17 complex. Formation of this complex depends on the level of Atg13 phosphorylation in which the complex 1 of TOR-TORC1 kinase (target of rapamycin complex 1) is involved. Inactivation of the TOR kinase complex leads to dephosphorylation of Atg13, causing an increase of its affinity to Atg1 and Atg17 and induction of the isolating membrane formation (Yang et al. 2010). The equivalent of the yeast Atg1-Atg13Atg17 complex is mammalian complex composed of the serine-threonine kinase ULK1/2 (Unc51-like kinase 1), a homologue of Atg1, the mAtg13 protein (mammalian Atg13), and FIP200, a mammalian homologue of Atg17 which stabilizes expression of and phosphorylates ULK1/2 (Hara et al. 2008).

To maintain stability and phosphorylation of the ULK1/2 kinase and mAtg, the presence of the Atg101 protein in the phagophore is necessary (Hosokawa et al. 2009a; Mercer et al. 2009). In mammalian cells, the autophagy induction depends on the activity of the complex 1 of mTOR kinase (mammalian target of rapamycin). Under physiological conditions, the mTORC1 complex, bound to the ULK1/2-mAtg13-FIP200 complex, is active and phosphorylates the S6K1 kinase. The mAtg13 protein is kept in the hypophosphorylated form, which results in its low affinity to the ULK1/2 kinase. Under starvation conditions or in the deficiency of growth factors, the mTORC1 complex is inactivated and dissociates from the above mentioned complex. This leads to the dephosphorylation of mAtg13 and subsequent activation of ULK1/2 which phosphorylates FIP200 and mAtg13. As a result, the autophagosome membrane formation is initiated (Jung et al. 2010; Mehrpour et al. 2010; Nakatogawa et al. 2009; Yang et al. 2010).

### Nucleation and elongation

At the early stage of the isolating membrane formation, i.e. during nucleation, the presence of the specific complex is required; this complex consists of class III phosphatidil-inositol kinase PI3K (a homologue of the yeast Vps34; vacuolar protein sorting 34), Beclin 1 (Atg6 in yeast), and serine kinase p150 (Vsp15 in yeast) (Kost et al. 2011). The involvement of PI3K in autophagy consists in its crucial role in phagophore elongation, as shown in mammalian cells (Yang et al. 2005; Yorimitsu and Klionsky 2005). The PI3K-Beclin 1-p150 complex, whose core is located in the phagophore, has an enzymatic activity causing production of phosphatidilinositole triphosphate (PIP3) that is indispensable at the stage of phagophore elongation and recruitment of next proteins from the Atg family (Kost et al. 2011). The complex activity, and production of PIP3, is strictly regulated by a battery of other proteins. The UVRAG (UV irradiation resistance-associated tumor suppressor gene) protein, a homologue of yeast Vsp38, is a positive regulator of the autophagosome maturation as it stimulates kinase activity of PI3K (Wong et al. 2011). On the other hand, proteins from the Bcl-2 family (B-cell leukemia/lymphoma-2) and the protein Ambra1 (activating molecule in Beclin1-regulated autophagy) are negative regulators. They bind Beclin 1 and block interactions with PI3K and formation of the complex (Fimia et al. 2007; Zhong et al. 2009; Wong et al. 2011).

In the next step of the isolating membrane elongation, there are two conjugation processes which require the involvement of two protein complexes. The first one is Atg5-Atg12-Atg16L, which requires Atg7 and Atg10 for its formation. Atg12 has to be activated by ATP-dependent formation of thioester bond with Atg7, to form an intermediate complex. Then, it is transferred to Atg10, also forming a thioester bond, and finally it is conjugated with Atg5 through an amide bond. This bond appears to be un-reversible, as no protease able to cut the Atg12-Atg5 complex could be found (Yang et al. 2005; Mariňo and López-Otín 2004).

At the later step, the Atg16L protein is attached to the Atg12-Atg5 protein, forming a covalent bond with Atg5. The Atg12-Atg5-Atg16L complex oligomerizes, forming structures that are used during elongation of the isolating membrane, which results in appearance of the pre-autophagosomal membrane (Yang and Klionsky 2010). It is possible that attachment of the complex may cause the membrane curvature. Initially, the proteins are attached uniformly on the membrane when it is being formed, however, as the phagophore is being elongated, they move towards its outer surface and then they dissociate when the autophagosome is complete. The second complex of proteins taking part in the synthesis of the autophagosomal membrane is Atg8-PE. Apart from Atg8 and phosphatidylethanolamine (PE), Atg3, Atg4 and Atg7 proteins participate in its formation. Activation of the Atg8 protein is initiated due to removal of the C-terminal amino acid, alanine, by the cysteine protease Atg4. This allows to form a thioester bond between Atg8 and Atg7 proteins. The Atg8 protein is transferred on the Atg3 protein, forming another thioester bond. Finally, the Atg8 protein forms a complex with PE through amide bond, thus, PE can be incorporated into the outer membrane of the autophagosome. This bond is reversible, contrary to the bond in the Atg12-Atg5 complex. After the fusion of autophagosome with lysosome, the amide bond is hydrolyzed by the Atg4 protease, and Atg8 dissociates into the cytoplasm (Mariňo and López-Otín 2004).

In mammalian cells, MAP1-LC3 is an equivalent of the yeast Atg8 protein. It was initially considered as a microtubule-associated protein (microtubule associated protein 1 light chain 3). This protein occurs in the cell in 3 forms: (i) pro-LC3, (ii) LC3-I, and (iii) LC3-II. During post-translational modification, pro-LC3 is transformed into LC3-I due to removal of 22 C-terminal amino acids by a mammalian homologue of the yeast Apg4 protease (Kirisako et al. 2005). The LC3-I protein remains in the cytoplasm, with exposed C-terminal glycine, until autophagy is initiated. Then, Atg7 and Atg3 proteins are attached through the C-terminal glycine, and subsequent attachment of the Atg12-Atg5-Atg16L complex results in incorporation of PE and creation of the LC3-II form (Reggiori and Klionsky 2005; Høyer-Hansen and Jäättelä 2007). LC3-II binds to both outer and inner membrane of the forming autophagosome (Yang et al. 2005; Kondo et al. 2005). This protein is released from PE to cytoplasm in the LC3-I form only after the fusion of the mature autophagosome with lysosome. A fraction of LC3-II bound with the inner membrane of autophagolysosome is degraded by lysosomal enzymes (Tanida et al. 2005). These processes cause formation of the autophagosome from the isolating membrane (phagophore), the structure containing a fragment of the cytoplasm together with some proteins and organelles. LC3-II is the only protein which binds specifically to the isolating membrane, autophagosome and autophagolysosome (Yang et al. 2005; Hayashi-Nishino et al. 2009; Carew et al. 2009). Conversion of LC3-I to LC3-II correlates with formation of autophagosome in the cell. In fact, the level of LC3-II is strictly correlated to number of autophagosomes in the cell, thus, it is the only known marker of the autophagy process (Tanida 2011; Sirdharan et al. 2011; Kost et al. 2011).

### Fusion of autophagosome with lysosome

After formation of the autophagosome, the outer membrane of autophagosome and lysosomal membrane fuse and form autophagolysosome (sometimes called autolysosome). In mammalian cells, this process is more complicated than in *S. cerevisiae.* Autophagosome fuses first with early and late endosomes which provide not only compounds to be degraded, but also factors required for the fusion between autophagosome and lysosome. It appears that endosomes lower the pH inside autophagosomes, creating favorable conditions for actions of lysosomal hydrolases (Glick et al. 2010). There are several proteins regulating the fusion, including LAMP-2 (Tanaka et al. 2000), monomeric GTP-ases (Rab7, Rab22, Rab24), Rubicon and proteins from the SNARE family (SNAP (Soluble NSF Attachment Protein) REceptor) (Yang et al. 2010). Mutations in the gene coding for the Rab7 protein impair the fusion of autophagosomes with late endosomes and lysosomes (Gutierrez et al. 2004; Yang et al. 2005). Proteins from UVRAG family enhance the Rab7 activity, which promotes such fusions, however, the same family negatively regulates autophagosome maturation when interacting with the Rubicon protein (Wong et al. 2011).

The fusion of autophagosome with lysosome requires also cytoskeleton elements. It was demonstrated that compounds which destabilize microtubules also inhibit autophagosome maturation. In cells treated with cytochalazine D, which blocks actin polymerization, a decrease in number of autophagosomes and autophagolysosmes was observed. Moreover, after treatment with nocodazole, which interferes with microtubule dynamics, the fusion of autophagosome with lysosome was blocked (Köchl et al. 2006). On the other hand, taxol, which stabilizes microtubules, caused an increase in efficiency of autophagolysosome formation (Mariňo and López-Otín 2004; Yang et al. 2005).

### Lysosomal degradation

Inside the autophagolysosme, there is an acid environment, which assures optimal pH for action of acid hydrolases which digest compounds enclosed inside this structure, together with its internal membrane. Thus, autophagolysosomes become single-membrane structures containing degraded compounds of the cytoplasm (Roy and Debnath 2010).

## Autophagy activation pathways

In the regulation of the autophagy process, there are several signaling pathways. They can be generally divided into mTOR-dependent and mTOR-independent ones. The mTOR protein is an evolutionarily conserved 289 kDa serine-threonine kinase. It plays roles not only in autophagy regulation but also controls transcription of genes and translation of proteins involved in microtubule dynamics. Moreover, it influences growth and proliferation of cells, as well as glucose metabolism (Pattingre et al. 2008). Through integration of intracellular signaling and growth factors, this kinase maintains the balance between protein biosynthesis and cell growth. It is also a sensor for energetic molecules in the cell, the ATP level, and the redox state (Roy and Debnath 2010).

**Fig. 1:**
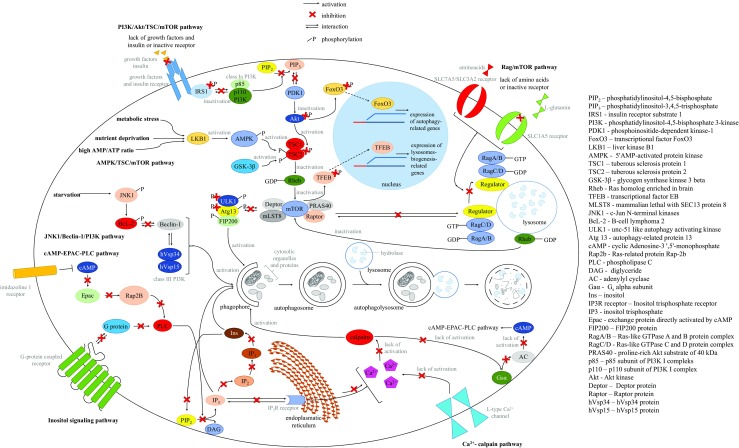
Pathways of autophagy stimulation. Detailed description is provided in the text

Among the autophagy stimulation pathways, the mTOR-dependent ones include: PI3K/Akt/TSC/mTOR, AMPK/TSC/mTOR, and Rag/mTOR pathways. Although the mTOR kinase is considered the main regulator of autophagy, there are also mTOR-independent pathways of autophagy activation. They include: Ca2+/calpain, inositol-dependent, cAMP/EPAC/PLC, and JNK1/Beclin-1/PI3K pathways. Both mTOR-dependent and mTOR-independent pathways are described below and summarized schematically in Fig. 1.

### The PI3K/Akt/TSC/mTOR pathway

The PI3K/Akt/TSC/mTOR pathway is initiated under conditions of a lack or low level of insulin or growth factors, which makes the insulin receptor inactive (Sarbassov et al. 2005; Massacesi et al. 2013). This results in a lack of phosphorylation of its substrate, the IRS1 protein, which in turn, cannot interact with the PI3K complex and activate it. Inactive PI3K complex is not able to promote the conversion of phosphatidilinositol-4,5-biphosphate (PtdIns(4,5)P2) to phosphatidylinositol-3,4,5-triphosphate (PtdIns(3,4,5)P3). The role of PtdIns(3,4,5)P3 is recruitment of the PDK1 kinase to the cellular membrane, however, deficiency of PtdIns(3,4,5)P3 results in a lack of activity of PDK1 that is not able to phosphorylate the Akt kinase (Sarkar 2013; Ravikumar et al. 2010). Therefore, Akt remains inactive, and no phosphorylation of the TSC complex, composed of the TSC1/2 heterodimer, is possible (Huang and Manning 2008). Dephosphorylated TSC complex has a GAP (GTPase-activating protein) activity which allows it to stimulate Rheb GTPase, and Rheb remains in the GDP-bound form. This Rheb form is not able to interact with the Raptor protein which is a component of the complex 1 of mTOR kinase (mTORC1). Thus, mTOR remains inactive and cannot interact with the ULK1-Atg13-FIP200 complex (Sarkar 2013), which results in a lack of phosphorylation of ULK1 and Atg13 proteins. This leads to the feedback regulation, as dephosphorylated ULK1 is activated which leads to phosphorylation of Atg13, FIP200 and ULK1 itself. In fact, this triggers formation of the autophagosome (Yang et al. 2005).

In addition to the above mechanism, due to inactivation of the Akt kinase, the FoxO3 transcription factor is not phosphorylated. As such, it migrates to the nucleus and stimulates expression of genes coding for proteins involved in the autophagy process, i.e. LC3 (autophagosome formation), Vsp34 (activation of the JNK1/Beclin-1/PI3K pathway), and ULK1 (induction of the PI3K/Akt/TSC/mTOR pathway) (Stitt et al. 2004). Moreover, inhibition of the mTOR kinase leads to dephosphorylation of TFEB (transcription factor EB). This transcription factor is also translocated to the nucleus and stimulates expression of genes involved in the formation of autophagosomes and lysosomal biogenesis (Vodicka et al. 2016; Roczniak-Ferguson et al. 2012).

### The AMPK/TSC/mTOR pathway

This pathway influences the autophagy activation by a sudden change in the energetic state of the cell, a lack of growth factors or metabolic stress (Meijer and Codogno 2007). AMPK is a cellular energy sensor, thus providing information to the cell about changes in the ATP/ADP ratio (Hardie 2007). Changes in this ratio lead to activation of the LKB1 kinase, resulting in phosphorylation, and thus activation of the AMPK kinase (Shaw et al. 2004). This allows phosphorylation of the TSC2 protein in the TSC complex. Interestingly, this AMPK-mediated phosphorylation unmasks another phosphorylation side in the target protein, which is then used to introduce another phosphate group by the GSK-3β kinase. One should note that under starvation conditions, the AMPK kinase phosphorylates TSC2 directly, without LKB1 involvement. Irrespective of the mechanism leading to TSC2 phosphorylation, this causes appearance of the GDP-bound Rheb form and inhibition of its interaction with mTOR, leading to autophagy induction (Inoki et al. 2003, 2006).

### The Rag/mTOR pathway

Activation of autophagy by the Rag/mTOR pathway depends on the availability of amino acids (Sarkar 2013). There are two receptors in the cell membrane responsible for detection of these compounds. The first one is the SLC1A5 (solute carrier 1A5) receptor, able to bind and transport L-glutamine from environment to the cell. Increased concentration of L-glutamine stimulates the heterodimeric bidirectional carrier, SLC7A5-SLC3A2, which removes it from the cell, but at the same time transports other amino acids into the cell (Nicklin et al. 2009). Under conditions of amino acid deficiency, or in the case of the dysfunction of the above mentioned receptors, these compounds cannot be caught by the complex of GTPases: Rag (Ras-related GTP-binding protein), regulator and v-ATPase, localized in the lysosomal membrane. Rag GTPases occur as heterodimers, composed of subunit A or B, connected to subunit C or D (Zoncu et al. 2011). In the presence of amino acids, the heterodimer is activated to form a conformation in which Rag A/B is bound to GTP, and Rag C/D is bound to GDP. In contrast, in the lack of amino acids, Rag A/B is bound to GDP and Rag C/D is bound to GTP (Kim and Kim 2016), and this conformation is inactive, thus, unable to interact with the Raptor protein that is a part of the mTORC1 complex. Therefore, the complex cannot bind to the lysosomal surface, and it is not activated by GDP-Rheb (Sancak et al. 2008). The inactive mTOR kinase cannot interact with the ULK1-Atg13-FIP200 complex, enabling initiation of the autophagosome formation (Hosokawa et al. 2009b).

### The Ca^2+^/calpain pathway

The Ca2^+^/calpain pathway is activated under conditions of any severe changes of physiological conditions. Another factor influencing this pathway is concentration of Ca^2+^ ions inside the cell, which activate calpains, proteins belonging to the family of cysteine proteinases (Goll et al. 2003). They can be activated not only by Ca^2+^ ions transported into the cell through the calcium channel, but also by the ions liberated from endoplasmic reticulum (Gordon et al. 1993; Williams et al. 2008; Ganley et al. 2011).

Autophagy induction by the Ca2^+^/calpain pathway can be stimulated by antagonists of Ca2^+^ canals type L (Williams et al. 2008; Zhang et al. 2007). They inhibit the inflow of the ions to the cell, thus, calpains are not activated. Generally, high Ca2^+^ levels and calpain activation negatively regulate the autophagosome formation and its fusion with lysosome (Sato-Kusubata et al. 2000). However, when the Ca2^+^ canals are closed, the level of Ca2^+^ ions is low, calpains are not activated, and the autophagy process can be initiated (Williams et al. 2008).

It is worth mentioning that the Ca2^+^/calpain pathway is connected to the pathway dependent on cAMP (described below). When nutrients are available and Ca2^+^ canals are open, active calpains stimulate dissociation of the α subunit of the G protein and its activation which, in turn, activates the AC (adenylate cyclase) protein to produce cAMP from ATP. In the deficiency of Ca2^+^ ions, this pathway is not activated, cAMP is not synthesized, which stimulates autophagy through the cAMP/EPAC/PLC pathway (Sato-Kusubata et al. 2000; Williams et al. 2008).

### The inositol-dependent pathway

One of autophagy activation pathways is a cascade of reactions dependent on the intracellular levels of inositol. This cascade is stimulated by blocking of the G protein-coupled receptor, which mediates activation of the phospholipase C (PLC). Under such conditions, inositol triphosphate (IP3) and diacylglycerol (DGA) are not formed from 4,5-phosphatidilinositol-bis-phosphate (PIP2) (Berridge 1993). Free inositol arises as a result of hydrolysis of two phosphate moieties from IP3 by inositol polyphosphatase (IPPase) and inositol-5’-phosphatase, and subsequent hydrolysis of inositol phosphate by inositol monophosphatase (IMPase) (Majerus 1992). When PIP2 cannot be converted to IP3, no free inositol is present which causes a lack of inhibition of autophagosomal membrane formation; this triggers the autophagy process.

There is a link between the inositol-dependent pathway and the Ca^2+^/calpaine pathway. Under normal physiological conditions, IP3 interacts with its receptor (IP3R) located in the endoplasmic reticulum which stimulates an increase in the level of Ca^2+^ ions in the cell and activation of calpains. In the absence of IP3, due to inhibition of the inositol-dependent pathway, the Ca^2+^/calpaine pathway is also impaired due to a lack of interaction with IP3R and low level of Ca^2+^ ions (Berridge 1993; Berridge et al. 2003).

### The cAMP/EPAC/PLC pathway

The autophagy is regulated by 3’5’adenosinemonophosphate (cAMP), independently from the mTORC1 kinase. cAMP is synthesized by adenylate cyclase from adenosine-5’-triphosphate (ATP) (Williams et al. 2008). Major activators of this pathways belong to agonists of imidazoline receptor (which acts to decrease the cAMP level). By activation of this receptor, these compounds cause a decrease in cAMP concentration in the cell (Williams et al. 2008). This results in a lack of EPAC (exchange protein directly activated by cAMP) activation, and resultant maintenance of the Rap2B protein in its inactive state (Gloerich and Bos 2010; Breckler et al. 2011). Under such conditions, phospholipase C (PLC) is not activated, and PIP2 cannot be converted to IP3 (see the inositol-dependent pathway, described above) (Sarkar et al. 2005). Both cAMP and IP3 are inhibitors of the phagophore formation, thus, impairment of the above described cascade results in autophagy activation.

### The JNK1/Beclin-1/PI3K pathway

The phosphoinositol kinase 3 (PI3K) complex consists of several class of enzymes. In the JNK1/Beclin-1/PI3K pathway, the crucial role is played by class III (PI3KC3) which include Beclin-1, pVps34, and pVps15 (Pattingre et al. 2008). Starvation conditions result in phosphorylation of the Bcl-2 protein by the stress-activated c-Jun-N-terminal protein kinase 1 (JNK1). This leads to inhibition of interaction between Bcl-2 and Beclin-1, and dissociation of the phosphorylated Bcl-2 form from the Bcl-2-Beclin-1 complex. Liberated Beclin-1 can interact with hVps34, which is a prerequisite to form the Beclin-1-hVps34-hVps15 complex. The latter complex directly stimulates formation of the autophagosome (Pattingre et al. 2005; Wei et al. 2008).

## Stimulation of autophagy as a therapeutic approach for treatment of metabolic neurodegenerative diseases

A large group of disorders in which autophagy induction might be profitable for treatment are metabolic brain diseases. Most of them is caused by the aggregation of improperly folded proteins which accumulate in neurons and cause their damage, leading to various severe psycho-motoric disorders. Pharmacologic induction of autophagy is one of the most promising approaches. An alternative pathway, the proteasomal degradation, is significantly less efficient in cells of patients due to ongoing proteasome damage by newly formatting protein aggregates (Zheng et al. 2016).

Studies on the therapeutic use of autophagy activators in neurodegenerative diseases are carried out in many laboratories around the world. However, researchers are still looking for compounds which not only stimulate degradation of accumulated, toxic macromolecules, but also are safe and suitable for the use in long-term use without severe adverse effects. The strategy of pharmacological stimulation of toxic macromolecules’ degradation is being tested using both cellular and animal models of neurodegenerative diseases, while due to relatively recent onset of such studies, there are only a few completed clinical trials, and most of them are either ongoing or at the stage of patients’ recruitment. In addition, new autophagy stimulators are being discovered which give promising results in experiments performed *in vitro* or with the use of animal models.

The most frequently used models of neurodegenerative disorders in studies on efficacy of autophagy stimulation are Huntington disease (a monogenic disorder whose etiology is well established), amyotrophic lateral sclerosis or ALS (a disease with predominance of one gene dysfunction, but with contribution of other factors), Alzheimer disease and Parkinson disease (disorders caused by multiple factors, but including inherited forms in which mutations in particular genes occur), and prion diseases (disorders which can be caused by a mutation, but prone to develop due to protein-protein interactions). The common feature of all these diseases is accumulation of misfolded proteins in neurons and a lack of effective treatment. Therefore, autophagy induction appears to be a promising potential therapeutic strategy. The above mentioned diseases are summarized briefly in Table 1.

**Table 1:** Neurodegenerative diseases which are model disorders in studies on autophagy inductors as potential therapeutics

Disease	Classification	Stored material	Cause of disease	Inheritance
Alzheimer disease (AD)	Proteinopathy (amyloidose, tauopathy)	β-amyloid; hyperphosphorylated tau protein	Mutation (familial form); unknown – perhaps multifactorial (sporadic form)	Autosomal dominant (familial form); multifactorial (sporadic form)
Parkinson disease (PD)	Proteinopathy (Syncleinopathies)	α-synuclein; parkin	Mutation (familial form); unknown – perhaps multifactorial (sporadic form)	Unclear; multifactorial (sporadic form)
Huntington disease (HD)	Proteinopathy	Huntingtin	Mutation	Autosomal dominant
Amyotrophic lateral sclerosis (ALS)	Proteinopathy	Superoxide dysmutase (SOD)	Mutation (familial form); unknown – perhaps multifactorial (sporadic form)	Unclear; multifactorial (sporadic form)
Creutzfeldt-Jakob disease, Gerstmann-Sträussler-Scheinker disease	Prion disease	PrP (prion protein)	Mutation (familial form); interactions between wild-type and misfolded proteins	Autosomal dominant (familial form); presence of the misfolded protein form

## Compounds activating autophagy and their therapeutic potential in treatment of metabolic brain diseases

### Rapamycin

One of the best known autophagy activators is rapamycin which inhibits the mTOR kinase activity. This compound is widely used as a drug inhibiting lymphocyte activation, thus, as an immunosuppressant it is employed in treatment of patients subjected to transplantations. Rapamycin binds to the cytosolic protein FKBP-12, and following formation of the tertiary complex with mTOR, the kinase activity of this protein is inhibited. As a result, the mTOR kinase substrate, 4EBP1, is not phosphorylated (Jacinto et al. 2004) which leads to destabilization of the mTOR-Raptor complex (Kim et al. 2002).

Rapamycin is used in studies on neurodegenerative diseases in both *in vitro* and *in vivo* models. Studies on cellular models gave promising results, indicating a possibility to enhance degradation of proteins which cause different disorders, including mutant huntingtin (mHTT) (Ravikumar et al. 2002; Sarkar et al. 2008) and alpha-synuclein (Webb et al. 2003). Nevertheless, more studies are being conducted with animal models. Effects of rapamycin on Huntington’s disease were tested in studies employing models of *Drosophila melanogaster* (Ravikumar et al. 2004; Sarkar and Rubinsztein 2008; Berger et al. 2006), zebrafish (Williams et al. 2008; Sarkar et al. 2011), and mice (Ravikumar et al. 2004). Animal experiments indicated a decrease in levels of mHTT aggregates, amelioration of neurodegeneration and improved animal behavior. Despite encouraging results of these studies, clinical trials with the use of rapamycin for treatment of HD have not been started yet.

Another disease in which rapamycin was tested using animal models is AD. Levels of beta-amyloid and hyperphosphorylated tau protein were determined as basic parameters considered in AD pathology. Experiments with AD mice overproducing the mutated gene coding for the tau protein indicated a highly elevated level of hyperphosphorylated form of this protein, while treatment with rapamycin caused its significant reduction and a decrease in number of neurofibrylar tangles (Ozcelik et al. 2013). Similar tendency was observed in mice producing toxic beta-amyloid, where rapamycin decreased the amount of this compound in the brain and improved the cognitive deficits (Spilman et al. 2010; Majumder et al. 2011; Zhang et al. 2017a; Caccamo et al. 2010).

In rapamycin-treated cellular models of PD, levels of alfa-synuclein aggregates were decreased due to stimulation of both lysosomal (autophagy) and proteasomal degradation (Webb et al. 2003).

Another example of the disease in which rapamycin was tested as a potential drug is Gerstmann-Sträussler-Scheinker syndrome, belonging to the group of prion diseases. Studies on the mouse model of this disease revealed prolonged life span, delayed symptoms and milder phenotype in animals treated with rapamycin (Cortes et al. 2012). On the other hand, different results were observed in the mouse model of ALS. Despite induction of autophagy, in rapamycin-treated mice, degeneration of motor neurons was enhanced relative to untreated controls and the life span was shorter (Zhang et al. 2011b). No significant differences were observed in the levels of aggregated superoxide dysmutase (SOD) between both groups of animals. The cause of enhanced neurodegeneration in mice treated with rapamycin remains unknown, but it seems unlikely to be due to toxicity of mutated SOD.

Despite many encouraging results of studies on rapamycin in cellular and animal models of neurodegenerative diseases, clinical trials have not been performed yet. Some doubts appeared due to the presence of adverse effects occurring in patients treated with this compound as an immunosuppressant. They include severe infections, hemolytic-uremic syndrome, cancer, leukopenia, and bone atrophy. Such adverse effects might be perhaps acceptable in a short-term treatment, for example in the transplantation procedures, however, in a long-term use, which is necessary in neurodegenerative diseases, they would be dangerous for patients.

### L-NG-Nitroarginine methyl ester

L-NAME (L-NG-Nitroarginine methyl ester) is an activator of the PI3K/Akt/TSC/mTOR pathway and mTOR-independent JNK1/Beclin-1/PI3K pathway. This compound inhibits formation of nitric oxide, which negatively regulates activity of the JNK1 kinase, leading to impairment of formation of the hVps34/Beclin 1 complex that is required for autophagosome formation. Thus, L-NAME-mediated deprivation of the NO level promotes creation of autophagosomes and enhances efficiency of autophagy. This mechanism has been employed in studies on cellular and animal models of HD (Sarkar et al. 2011). Recent studies on cancer cells indicated that L-NAME induces also another pathway of autophagy stimulation, namely the PI3K/Akt/TSC/mTOR pathway (Zhu et al. 2017). However, determination of relevance of this mechanism in neurodegenerative diseases requires further studies. L-NAME has been tested in studies on AD. Intracranial injection of this compound resulted in improvement of memory and learning of AD mice (in the Morris water maze test) and an increase of the level of autophagy markers, relative to untreated animals (Shariatpanahi et al. 2015).

### Trehalose

Trehalose activates both mTOR-dependent and mTOR-independent pathways of autophagy stimulation. It activates the AMPK/TSC/mTOR pathway, however, it also enhances expression of the gene coding for beclin, thus, it acts by stimulation of the JNK1/Beclin-1/PI3K pathway, which is an mTOR-independent mechanism of autophagy activation (Vidal et al. 2014). Translocation of the FoxO transcription factor, a substrate for the Akt kinase (in the mTOR-dependent pathway) has been demonstrated (DeBosch et al. 2016). This indicates that trehalose, similarly to L-NAME, is an inductor of at least two pathways leading to autophagy stimulation. This is supported by results indicating involvement of trehalose in the regulation of the AMPK-dependent pathway (DeBosch et al. 2016).

Promising results were obtained in studies on cellular models of PD in which induction of autophagy (Zhao et al. 2017) or proteasomal pathway of protein degradation (Lan et al. 2012) is accompanied with a decrease in the level of quickly aggregating form of A53T-mutated alpha-synuclein. In addition, the cells producing this toxic protein were protected against apoptosis.

In studies on the cellular AD model, it was found that the level of endogenous tau protein was reduced and the toxicity caused by formation of aggregates was alleviated after treatment with trehalose (Krüger et al. 2012). It was, therefore, suggested, that this compound might be effective not only in treatment of AD but also other tauopathies. Studies on animal models demonstrated a decreased number of neurons with tau protein aggregates and lower numbers of these aggregates in cells, as well as improved viability of neurons (Schaeffer et al. 2012) as a results of treatment with trehalose. Another study with similar approach indicated improvement of motor functions of animals and alleviation of fear (Rodríguez-Navarro et al. 2010).

Decreased quantity of mHTT aggregates and significant improvement of behavior were observed in analogous studies with HD mouse model (Perucho et al. 2016). When trehalose was tested in cellular models of ALS, autophagy-dependent degradation of SOD1 aggregates led to increased viability of neurons. These results were corroborated by studies on the animal model of ALS, in which prolongation of the life span was observed (Castillo et al. 2013).

### Resveratrol

Resveratrol is another stimulator of the AMPK/TSC/mTOR pathway. It activates the AMPK kinase which leads to stimulation of the autophagy process (Burkewitz et al. 2014). Efficiency of this compound in removal of toxic protein has been tested in various diseases. Resveratrol has induced autophagy and enhanced degradation of mHTT in the cellular model of HD, in which neuroblastoma SH-SY5Y line was used. Moreover, level of the Atg4 (which is decreased in mHTT-accumulating cells) normalized (Vidoni et al. 2017). This compound has also been used in the 3-nitropropionic acid (3-NPA)-induced rat model of HD. However, it is worth to note that in such animals, neurodegeneration occurs due to changes in mitochondrial metabolism which leads to (i) production of reactive oxygen species, (ii) changes in cellular energetics, and (iii) induction of apoptosis. As a consequence, hypo- and hyper-motoric changes appear which resemble symptoms of HD. Nevertheless, chorea, dyskinesis and dystony never occur in 3-NPA-treated murine models, while they are the most characteristic symptoms in humans. Moreover, this compound does not cause appearance of mHTT, the primary cause of the disease, thus, only secondary effects can be tested (Túnez et al. 2010). Hence, mechanisms of autophagy-mediated degradation of mHTT could not be studied in this model, and the tests included only anti-oxidant properties of resveratrol, which caused an increase in the level of glutathione and a decrease in levels of nitrites, as well as less efficient peroxidation of lipids. Thus, results of memory and motoric tests were better in treated animals than in controls (Kumar et al. 2006). On the other hand, experiments with transgenic HD mice have also been performed. It was suggested that activation of SIRT1 (mammalian sirtuin) by resveratrol increases viability of neurons (Ho et al. 2010). However, this compound did not affect changes in the striatum, motor functions and life span of mice.

To test effects of resveratrol on PD, mouse neuroblastoma cell lines (N2a cells) were treated with this compound in combination with β-cyclodextrin. Number of alpha-synuclein aggregates decreased and viability of the cells increased (Gautam et al. 2017). In animals, PD can be induced by MPTP (1-methyl-4-phenyl-1,2,3,6-tetrahydroxypyridine), which is converted to MPDP^+^ (1-methyl-4-phenyl-2,3-dihydropyridinium), and then to the active metabolite MPP^+^ (1-methyl-4-phenyl-pyridinium) by astrocytes and acts as an inhibitor of complex I of the mitochondrial electron transport system. This active toxin is catched by dopaminergic neurons in striatum and leads to their degeneration (Porras et al. 2012). When resveratrol was administered to mice before MPTP, a protection against neurodegeneration was observed, dopamine was kept at normal levels, and animal behavior was significantly less changed relative to animals treated solely with the toxin. Molecular studies indicated that SIRT1 is activated by resveratrol which leads to LC3 deacetylation and induction of autophagy. As a result, number of alfa-synuclein decreased in dopaminergic cells (Guo et al. 2016). Other tests, performed with the use of the rat model, suggested an anti-oxidative mechanism of resveratrol action, since the red-ox balance has been re-established, endoplasmic reticulum stress was alleviated, and expression of genes coding for caspases was impaired, which might protected cells against apoptosis (Gaballah et al. 2016).

### Calcium canal antagonists

Antagonists of calcium canals, which also activate the Ca^2+^/calpain pathway, are relatively often tested in metabolic brain diseases. The list of such compounds include: latrepirdine, verapamil, loperamide, nitrendipine, nilvadipine, nimodipine, amiodarone, niguldipine, nicardipine, pimozide, penitrem A, fluspirilene, and trifluoperazine. When the calcium channel is blocked, the intracellular calcium level drops rapidly, thus, calpains are inactivated which stimulates autophagosome formation. Such compounds were tested in cellular models of HD, and it was found that they caused reduction of the mHTT level (Zhang et al. 2007; Williams et al. 2008).

Latrepiridine was tested in the cellular model of AD, and decreased levels of calcium ions were correlated with increased viability of cells (Lermontova et al. 2001). However, in that work, autophagy was not suggested as a mechanism leading to such improvement. Experiments with animal models of AD indicated decreased levels of beta-amyloid deposits in the brain and increased levels of autophagy markers in latrepiridine-treated mice (Bharadwaj et al. 2013). In similar experiments, improvement in cognitive tests was reported (Lermontova et al. 2000). However, a clinical trial phase III with this compound as a potential anti-AD drug, gave negative results (Chau et al. 2015; Sweetlove 2012; http://www.alzforum.org/news/research-news/dimebon-disappoints-phase-3-trial?id=2387). Similarly, phase III clinical trial with HD patients failed to demonstrate improvement after treatment with latrepiridine (https://www.genengnews.com/gen-news-highlights/phase-iii-failure-leads-medivation-and-pfizer-to-ditch-dimebon-for-huntington-disease/81244981/).

Verapamil has been tested in experiments with a HD mouse model. This drug caused improvement in motoric activity and keeping balance by animals (Kalonia et al. 2011). Although verapamil has not been tested in clinical trials for HD, its efficacy was assessed in treatment of ALS patients. However, 5-month treatment did not result in improvement of the disease parameters (Miller et al. 1996b).

When nitrendipine and nilvadipine were studied using AD models, inhibition of beta-amyloid accumulation *in vitro* and its enhanced degradation *in vivo*, accompanied with memory improvement in mice, were observed (Paris et al. 2011). Interestingly, quite similar results were obtained during experimental therapy of AD patients when nilvadipine and nimodipine prevented the progression of congnitive problems (Nimmrich and Eckert 2013). On the other hand, in the clinical trial phase III with ALS patients, nimodipine appeared ineffective and many adverse effects were reported, including diarrhea, nausea, and lightheadedness (Miller et al. 1996a).

Amiodarone has been tested mainly as a potential anti-AD drug. Experiments were conducted *in vitro* and *in vivo* (with the Guinea pig model). Amiodarone was used as a compound which elevates pH, thus, secretases that cut the APP protein (the amyloid precursor) and require acidic environment, were inactivated. Thus, the level of amyloid decreased, however, a mechanism involving stimulation of autophagy was not considered, though the authors suggest that elevation of pH is perhaps not the only way of action of the tested compound (Mitterreiter et al. 2010).

Pimozide, is already used in medicine for treatment of schizophrenia and psychotic disorders (Mothi and Sampson 2013). However, its positive effects were observed also in HD. Experimental therapy with a low number of patients indicated an improvement in hyperkinesia (Girotti et al. 1984). Studies with the mouse model of AD which overproduces hyperphosphorylated tau protein, indicated that intraperitoneal administration of pimozide resulted in reduction of aggregates of this protein and to memory improvement in animals (Kim et al. 2017). Interestingly, when considering a possible molecular mechanism of pimozide action, the authors did not consider the calcium channel-dependent pathway. On the contrary, they have detected an increased level of phosphorylation of AMPK and ULK1 kinases, and inhibition of the mTOR kinase activation. They have suggested that pimozide may stimulate as yet unknown pathway of autophagy induction which is dependent on AMPK-ULK interactions, with no involvement of mTOR (Kim et al. 2017). One case of an AD patient treated with pimozide for 5 weeks has been described, and alleviation of dementia was noted; the effects remained unchanged for next 9 months (Renvoize et al. 1987). Despite these results, no clinical trial with this compound was reported to date.

Trifluoperazine has been tested as a potential anti-HD and anti-AD drug. However, it was suggested that this compound may inhibit apoptosis, and stimulation of autophagy has not been considered (Lauterbach 2013). Studies with patients included only a very limited number of individuals, though the results were quite encouraging (Stokes 1975). On the other hand, trifluoperazine was widely tested for treatment of AD. However, a clinical trial with this compound indicated that life span of treated patients was shortened by 12 months relative to untreated controls (Ballard et al. 2009). In another clinical trial, no improvement of the disease symptoms could be find (Ballard et al. 2008). Interesting studies were conducted on a PD mouse model, characterized by moderate expression of the gene coding for synucleine, thus the time of death of neurons could be determined precisely. It was found that non-induced autophagy is impaired by accumulated synuclein, while trifluoroperazine-induced autophagy causes a delay in neurons’ death (Höllerhage et al. 2014). When screening for anti-PD compounds was conducted using a zebrafish model, trifluoperazine was identified as a molecule preventing the loss of neurons. It was demonstrated that apart from blocking calcium channels, trifluoperazine stimulated translocation of the transcription factor EB (TFEB; a master regulator for lysosomal biogenesis that also activates autophagy, when present in the nucleus in a non-phosphorylated form, by enhancing transcription of relevant genes) which is a substrate for the mTOR kinase. Therefore, this compound is another factor which can stimulate autophagy through more than one pathway (Zhang et al. 2017b).

Studies on other blockers of calcium channels (loperamide, niguldipine, nicardipine, panitrem A, and fluspirilene) as anti-neurodegenerative agents were terminated after *in vitro* studies (Zhang et al. 2007; Williams et al. 2008) due to adverse effects reported in the meantime by researchers using them for treatment of other diseases. These adverse effects included: constipation, dizziness, nausea, paralytic ileus, angioedema, anaphylaxis reactions, toxic epidermal necrolysis, Stevens-Johnson syndrome, erythema multiform, urinary retention, and heat stroke.

### Calpastatin

Calpastatin inhibits activities of calpains, thus, inhibition of autophagosome formation is abolished. Overproduction of calpastatin induced autophagy decreased levels of mHTT, improved motor functions, and delayed appearance of other symptoms in the mouse model of HD (Menzies et al. 2015). Importantly, prolonged administration of calpastatin did not cause any severe adverse effects in animals. In studies on the cellular AD model, an inhibitor of histone deacetylase, trichostatin A, which also increases production of calpastatin, caused an increase in viability of cells (Seo et al. 2013). These results are in agreement with observations that silencing of expression of calpastatin-encoding gene causes changes in cytoskeleton and lowers cell viability (Rao et al. 2008). Moreover, long-term activation of calpains causes overstimulation of many proteases, which leads to degradation of a number of cellular substrates, including cytoskeleton elements and membrane receptors involved in homeostasis maintenance. When calpastatin is overproduced, such effects can be diminished (Schoch et al. 2013). Overexpression of the calpastatin gene in the mouse model of PD resulted in reduction of the number of alpha-synuclein aggregates and improved signal transduction through synapses (Diepenbroek et al. 2014).

### Minoxidil

Minoxidil activates potassium channels which prevents the transport of calcium ions into the cell, leading to calpains inactivation and enhanced autophagy (Renna et al. 2010). When cellular models of PD and HD were investigated, minoxidil induced the autophagy process and a decrease in levels of alpha-synuclein and mHTT was observed which was accompanied with increased cell viability (Williams et al. 2008). However, this compound has not been tested yet in animal models.

### Lithium

Lithium is tested as a potential drug for many diseases affecting central nervous system (CNS). This agent inhibits activity of inositol monophosphatase, decreasing the level of inositol and IP3 which allows formation of the autophagosome membrane (Sarkar et al. 2005). However, lithium negatively regulates also activity of another enzyme, GSK-3β, causing stimulation of the mTOR kinase and autophagy inhibition. Therefore, it was proposed to combine the use of lithium and rapamycin (an inhibitor of mTOR). This approach appeared significantly more effective than the use of each component separately (Sarkar et al. 2008). However, in studies with the 3-NPA-induced HD rat model, treatment with LiCl for 8 days caused an increase in pathological changes in the brain (Milutinović 2016). On the other hand, it is worth remaining that 3-NPA does not cause the appearance of mHTT aggregates, thus, this is not an adequate model for testing potential drugs which might activate autophagy. In such studies, genetic models of mHTT would be much more relevant. It was also reported that lithium causes a decrease in the level of histone deacetylase (HDAC1) which is correlated with effective degradation of mHTT (Wu et al. 2013). Lithium has also been used in experimental therapy in which 3 patients suffering from HD were involved. In one patient, some neurological parameters were improved, but no changes in chorea could be observed. The second patient responded with improvement in chorea with no neurological changes. In the third patient, stabilization of all symptoms, but no improvement, was noted. Nevertheless, all these patients received also other drugs, including carbamazepine, which makes interpretation of the results very difficult (Danivas et al. 2013). Other clinical trials with HD patients also did not give conclusive results regarding efficacy of lithium due to extremely different responses of various persons (Scheuing et al. 2014).

When mice overproducing hyperphosphorylated tau protein were treated with lithium, a significant improvement in behavior and cognitive functions was observed, levels and phosphorylation of tau decreased, as did efficiency of beta-amyloid formation, and levels of autophagy markers increased (Shimada et al. 2012; Zhang et al. 2011a). Efficacy of lithium in the mouse model of PD was tested in a combined therapy with valproic acid. Improvement in behavior and an increase in the number of dopaminergic neurons were evident. Deprivation of dopamine and its metabolite, dihydroxyphenyloacetic acid, was less pronounced than in untreated animals (Li et al. 2013). Analogous combination of drugs was tested in the mouse model of HD. Treated animals expressed improvement in motoric functions and memory (as tested in the Morris water maze). Reduction of the level of mHTT aggregates and less pronounced loss of neurons in striatum were observed. Interestingly, expression of genes coding for proteins involved in mitochondrial metabolism, antioxidative response, apoptosis and anti-inflammatory reactions were significantly modulated (Linares et al. 2016). These results indicate the broad spectrum of biological activities of lithium and valproic acid, as suggest that a complex network of processes is involved in the pathogenesis of HD.

### Valproic acid

Valproic acid inhibits activity of myo-inositol-1-phosphate synthase, one of enzymes involved in the metabolism of inositol (Shaltiel et al. 2004), thus, causing a decrease of the level of the latter compound and activation of autophagy. Combination of valproic acid and lithium was tested in clinical trials with HD patients. However, in most cases either a lack of effects or only stabilization of symptoms (with no improvement) were observed (Scheuing et al. 2014).

AD model cells were treated with valproic acid, and no changes with the total amount of beta-amyloid were demonstrated while level of beta-amyloid oligomers (which are suggested to be more toxic) decreased and level of monomers increased, relative to untreated control cells (Williams and Bate 2018). This may suggest that beta-amyloid oligomers are converted to monomers in valproic acid-treated cells. Streptozotocin (STZ)-induced rat model of AD has been used in *in vivo* studies. Intraventricular injection of STZ provokes neurodegeneration and accumulation of beta-amyloid and hyperphosphorylated tau protein, thus, mimicking the sporadic form of AD. Decreased levels of acetylcholine and neprylysine, and increased activity of acetylcholinesterase cause additionally enhanced neurodegeneration and cognitive defects. Treatment with valproic acid resulted in prevention of cognitive deficits and normalization of levels and activities of neurotransmitters (Sorial and El Sayed 2017). Using another animal model of AD, transgenic mice expressing a mutated *APP* gene, effects of valproic acid in males and females were compared. Decreased levels of amyloid plaques were more pronounced in males than in females, while number of synaptic vesicles were similar in both genders. On the other hand, neurodegeneration was prevented more efficiently in males (Long et al. 2016).

Cellular models of PD were used to investigate the mechanism of action of valproic acid. This compound caused reduction of levels of proapoptotic proteins and ROS, while autophagy inhibitors diminished these effects, indicating a crucial role of this process in valproic acid-mediated improvement in PD cellular phenotypes (Zhang et al. 2017c). Other *in vitro* studies were based on the use of murine neurons treated with human beta-amyloid. Defects in synaptic proteins and neurotransmitter transporting vesicles were observed. These effects were alleviated by addition of valproic acid into the cell culture. A mechanism has been proposed in which this compound negatively regulates cytoplasmic phospholipase A2 (cPLAS2), whose overactivity correlates with neurodegeneration. Autophagy has been suggested as an additional mechanism of the observed changes in cells (Williams and Bate 2016).

Controversial results were obtained in studies on prion disease. Early studies suggested that valproic acid causes an increased accumulation of PrP in neuroblastoma cells and model cells for the disease. However, administration of valproic acid to Chinese hamsters infected with prions did not cause any effects on the course of the disease (Shaked et al. 2002). Other studies performed with cellular models did not confirm effects of valproic acid on the levels of PrP (Legendre et al. 2007).

### Carbamazepine

Mechanism of action of carbamazepine is similar to that by valproic acid and lithium (Williams et al. 2002). A decrease in the inositol level arises from deprivation of PIP2 and IP3 (Schiebler et al. 2015). Studies with the mouse model of AD indicated that carbamazepine improved learning abilities and memory, which was correlated with decreased number of amyloid plaques (Li et al. 2013; Zhang et al. 2017a). Apart from stimulation of mTOR-independent pathway of autophagy activation, carbamazepine inhibited the mTOR kinase activity. Therefore, it is another example of autophagy stimulation by more than one molecular mechanism (Li et al. 2013).

Since carbamazepine is known as an analgesic, anticonvulsant and antiepileptic drug, it has been used for treatment of HD patients (Danivas et al. 2013). It was proposed that its mechanisms of action is related to blocking calcium channels which cause inhibition of glutamate liberation (Kawata et al. 2001). Intriguingly, autophagy was not considered as a mechanism by which carbamazepine improves symptoms of HD.

An interesting case report has been published in which carbamazepine was administered to a patient suffering from hypertension, myocardial infraction, and atrial fibrillation. When high doses of the drug were used (as the patient became resistant to lower doses), many adverse effects were noted, including memory deficits, confusion, psychomotor slowness, hypersomnia, dysphasia, and postural instability with falls. The patient’s condition was continuously deteriorating. Psychological tests indicated attentional deficits, perseverations, severe non-fluent aphasia with paraphasias, and constructional apraxia. The EEG results were similar to those found in patients suffering from Creutzfeldt–Jakob disease. After cessation of treatment with carbamazepine both EEG results and patient’s conditions improved considerably. Cognitive deficit and motor dysfunctions normalized. Thus, it was concluded that carbamazepine caused Creutzfeldt–Jakob disease-like symptoms (Horvath et al. 2005).

### Clonidine

Clonidine binds and activates the imidazoline receptor, which leads to a decrease in the level of cAMP in cells (Williams et al. 2008). However, it appears that there is an additional mechanism of action of this compound, namely activation of potassium channels which causes a decrease in concentration of calcium ions in the cytoplasm (Murphy and Freedman 2001). Clonidine was used as one of compounds activating autophagy in the screening for a potential drug for HD and PD. It was effective in reducing amounts of synuclein and mHTT in cells (Williams et al. 2008). *In vivo* experiments were performed with reserpine-treated rat model of Parkinson's disease. Following injection of reserpine, severe akinesis was observed which could be prevented by previous treatment with clonidine (Hill and Brotchie 1999). However, stimulation of autophagy was not considered as a potential mechanism of action of this drug. In PD potential therapies concentrate on inhibition of movements while patients suffer also from cognitive deficits and mood swings. When clonidine, as an agonist of adrenergic receptor alpha-2, was tested as a potential drug at early phase of PD in a monkey model (*Macaca fasicularis*), it was found that the treatment caused improvement in concentration and memory (Schneider et al. 2010).

Models of memory deficits were also used in studies on clonidine. In murine models, the symptoms were induced by administration of NMDA (N-methyl-D-aspartate) antagonist MK-801 or by excitotoxic hippocampal damage. Clonidine ameliorated symptoms caused by MK-801, but did not change behavior of rats in which hippocampus was damaged by excitotoxic agents (Bardgett et al. 2008).

The only studies on the use of clonidine in prion disease were performed with the yeast model. However, no significant effects on the level of PrPSC could be observed (Tribouillard-Tanvier et al. 2008).

### Rilmenidine

Rilmenidine induces the autophagy proces through the cAMP/EPC/PLC pathway. Similarly to clonidine, it binds and activates the imidazoline receptor. It was tested in experiments with cellular HD and PD models, and caused a decrease in levels of mHTT and alpha-synuclein (Williams et al. 2008). In the mouse model of HD, reduction of mHTT levels was also observed but number of aggregates remained unchanged. Although rilmenidine could not prevent the body weight loss, it corrected the muscle parameters and general condition of the organism (Rose et al. 2010). Although a clinical study with 18 HD patients has been conducted, only 12 patients completed this trial. Some cognitive parameters and motor functions were improved (Underwood et al. 2017); however, a study with significantly higher number of patients is necessary to make solid conclusions.

Cellular and animal models of ALS were used to study effects of rilmenidine in this disease. A decrease in mutant SOD1 level was observed in cells in which macroautophagy and mitophagy were also evident. Similarly, administration of this drug to mice suffering from ALS resulted in autophagy induction in motor neurons. Unexpectedly, enhanced degeneration of these neurons was observed under these conditions. Moreover, accumulation of SOD aggregates and a decrease in number of mitochondria occurred in treated animals, and correlated with more severe symptoms relative to untreated mice. It was suggested that too extensive mitophagy could be responsible for these effects (Perera et al. 2017).

### Dideoxyadenosine

Dideoxyadenosine (2’5’ddA) inhibits the activity of adenylate cyclase which leads to a rapid decrease in the cAMP level in cells, and subsequent stimulation of autophagy. In studies with a cellular model of HD, treatment with 2’5’ddA resulted in decreased levels of mHTT and its aggregates (Williams et al. 2008). No reports were published on the use of this compound in experiments with animal models.

### SMER28

SMER28 appears to induce autophagy through both PI3K/Akt/TSC/mTOR and JNK1/Beclin-1/PI3K pathways (Sarkar et al. 2011). When cellular model of AD was employed, treatment with SMER28 prevented formation of beta-amyloid aggregates in a dose-response mode (Shen et al. 2011). When production of Beclin-1 was impaired, effects of SMER28 were diminished. Similar effects were observed in experiments with silencing of expression of the gene coding for ULK kinase. Therefore, SMER28 appears to stimulate mTOR-dependent and mTOR-independent mechanisms of autophagy activation pathways (Tian et al. 2011).

## Concluding remarks

There are various possibilities to induce autophagy. In this review, mechanisms of these pathways were discussed to indicate how this process can be stimulated, which is in contrast to most other review article that described mechanisms of negative regulations (in fact autophagy is regulated by various inhibitors). Stimulation of autophagy has been considered as a strategy for treatment of various neurodegenerative diseases. Although there are many encouraging results obtained in experiments with cellular and animal models (described in this review in particular sections presenting various ways of autophagy stimulation), specific treatments of metabolic brain diseases are not yet available. One of the most important problem is appearance of severe adverse effects when strong autophagy stimulators are tested. Such effects were observed for rapamycin, nimodipine, loperamide, niguldipine, nicardipine, panitrem A, fluspirilene, calpastatin, and carbamazepine. Therefore, a compound which activates this process but is also safe in the long-term use is highly desirable. In this light it is worth mentioning that genistein (5, 7-dihydroxy-3- (4-hydroxyphenyl)-4*H*-1-benzopyran-4-one), a natural isoflavone, has been demonstrated recently to decrease levels of mutant huntingtin and to reduce number and size of aggregates of this toxic protein in the cellular model of HD by autophagy stimulation (Pierzynowska et al. 2018). This isoflavone could alleviate lysosomal storage of glycosaminoglycans in vitro and in vivo (in visceral organs and in the brain), and correct animal behavior in various models of mucopolysaccharidosis type I, II and III, a neurodegenerative metabolic disease (Piotrowska et al. 2006; Friso et al. 2010; Malinowska et al. 2009, 2010). Genistien has been demonstrated to be safe for a long-term use (over 1 year) at the dose as high as 150 mg/kg/day (Kim et al. 2013). Therefore, it may be considered as a promising agent for development of an effective and safe therapeutics for treatment of neurodegenerative diseases by stimulation of autophagy.

Interestingly, one compound is sometimes able to activate the autophagy process by both mTOR-dependent and mTOR-independent pathways. Such molecules (exemplified by L-NG-nitroarginine methyl ester, trehalose, pimozide or trifluoperazine) are often very effective in removing toxic protein aggregates from cells. It is worth noting that there are links between different mTOR-dependent pathways, which may enhance effects of certain activators of autophagy. Although no such links were discovered between mTOR-dependent and mTOR-independent pathways, the existence of compounds that stimulate autophagy by both these mechanisms might suggest a possibility that there are some cross-talks between molecules involved in both types of pathways.

## References

[CR1] Ballard C, Lana MM, Theodoulou M, Douglas S, McShane R, Jacoby R, Kossakowski K, Yu LM, Juszczak E, Investigators DARTAD (2008). A randomised, blinded, placebo-controlled trial in dementia patients continuing or stopping neuroleptics (the DART-AD trial). PLoS Med.

[CR2] Ballard C, Hanney ML, Theodoulou M, Douglas S, McShane R, Kossakowski K, Gill R, Juszczak E, Yu LM, Jacoby R, investigators DART-AD (2009). The dementia antipsychotic withdrawal trial (DART-AD): long-term follow-up of a randomised placebo-controlled trial. Lancet Neurol.

[CR3] Bardgett ME, Points M, Ramsey-Faulkner C, Topmiller J, Roflow J, McDaniel T, Lamontagne T, Griffith MS (2008). The effects of clonidine on discrete-trial delayed spatial alternation in two rat models of memory loss. Neuropsychopharmacology.

[CR4] Berger Z, Ravikumar B, Menzies FM, Oroz LG, Underwood BR, Pangalos MN, Schmitt I, Wullner U, Evert BO, O'Kane CJ, Rubinsztein DC (2006). Rapamycin alleviates toxicity of different aggregate-prone proteins. Hum Mol Genet.

[CR5] Berridge MJ (1993). Inositol trisphosphate and calcium signalling. Nature.

[CR6] Berridge MJ, Bootman MD, Roderick HL (2003). Calcium signalling: dynamics, homeostasis and remodelling. Nat Rev Mol Cell Biol.

[CR7] Bharadwaj PR, Bates KA, Porter T, Teimouri E, Perry G, Steele JW, Gandy S, Groth D, Martins RN, Verdile G (2013). Latrepirdine: molecular mechanisms underlying potential therapeutic roles in Alzheimer's and other neurodegenerative diseases. Transl Psychiatry.

[CR8] Breckler M, Berthouze M, Laurent AC, Crozatier B, Morel E, Lezoualc’h F (2011). Rap-linked cAMP signaling Epac proteins: compartmentation, functioning and disease implications. Cell Signal.

[CR9] Burkewitz K, Zhang Y, Mair WB (2014). AMPK at the nexus of energetics and aging. Cell Metab.

[CR10] Caccamo A, Majumder S, Richardson A, Strong R, Oddo S (2010). Molecular interplay between mammalian target of rapamycin (mTOR), amyloid-beta, and Tau: effects on cognitive impairments. J Biol Chem.

[CR11] Carew JS, Medina EC, Esquivel JA, Mahalingam D, Swords R, Kelly K, Zhang H, Huang P, Mita AC, Mita MM, Giles FJ, Nawrocki ST (2009). Autophagy inhibition enhances vorinostat-induced apoptosis via ubiquitinated protein accumulation. J Cell Mol Med.

[CR12] Castillo K, Nassif M, Valenzuela V, Rojas F, Matus S, Mercado G, Court FA, van Zundert B, Hetz C (2013). Trehalose delays the progression of amyotrophic lateral sclerosis by enhancing autophagy in motoneurons. Autophagy.

[CR13] Chau S, Herrmann N, Ruthirakuhan MT, Chen JJ, Lanctôt KL (2015). Latrepirdine for Alzheimer's disease. Cochrane Database Syst Rev.

[CR14] Cortes CJ, Qin K, Cook J, Solanki A, Mastrianni JA (2012). Rapamycin delays disease onset and prevents PrP plaque deposition in a mouse model of Gerstmann-Sträussler-Scheinker disease. J Neurosci.

[CR15] Cuervo AM (2004). Autophagy: many paths to the same end. Mol Cell Biochem.

[CR16] Danivas V, Moily NS, Thimmaiah R, Muralidharan K, Purushotham M, Muthane U, Jain S (2013). Off label use of lithium in the treatment of Huntington's disease: A case series. Indian J Psychiatry.

[CR17] DeBosch BJ, Heitmeier MR, Mayer AL, Higgins CB, Crowley JR, Kraft TE, Chi M, Newberry EP, Chen Z, Finck BN, Davidson NO, Yarasheski KE, Hruz PW, Moley KH (2016). Trehalose inhibits solute carrier 2A (SLC2A) proteins to induce autophagy and prevent hepatic steatosis. Sci Signal.

[CR18] Diepenbroek M, Casadei N, Esmer H, Saido TC, Takano J, Kahle PJ, Nixon RA, Rao MV, Melki R, Pieri L, Helling S, Marcus K, Krueger R, Masliah E, Riess O, Nuber S (2014). Overexpression of the calpain-specific inhibitor calpastatin reduces human alpha-Synuclein processing, aggregation and synaptic impairment in [A30P] αSyn transgenic mice. Hum Mol Genet.

[CR19] Dong H, Czaja MJ (2011). Regulation of lipid droplets by autophagy. Trends Endocrinol Metab.

[CR20] Fimia GM, Stoykova A, Romagnoli A, Giunta L, Di Bartolomeo S, Nardacci R, Corazzari M, Fuoco C, Ucar A, Schwartz P, Gruss P, Piacentini M, Chowdhury K, Cecconi F (2007). Ambra1 regulates autophagy and development of nervous system. Nature.

[CR21] Friso A, Tomanin R, Salvalaio M, Scarpa M (2010). Genistein reduces glycosaminoglycan levels in a mouse model of mucopolysaccharidosis type II. Br J Pharmacol.

[CR22] Gaballah HH, Zakaria SS, Elbatsh MM, Tahoon NM (2016). Modulatory effects of resveratrol on endoplasmic reticulum stress-associated apoptosis and oxido-inflammatory markers in a rat model of rotenone-induced Parkinson's disease. Chem Biol Interact.

[CR23] Ganley IG, Wong PM, Gammoh N, Jiang X (2011). Distinct autophagosomal–lysosomal fusion mechanism revealed by thapsigargin-induced autophagy arrest. Mol Cell.

[CR24] Gautam S, Karmakar S, Batra R, Sharma P, Pradhan P, Singh J, Kundu B, Chowdhury PK (2017). Polyphenols in combination with β-cyclodextrin can inhibit and disaggregate α-synuclein amyloids under cell mimicking conditions: A promising therapeutic alternative. Biochim Biophys Acta.

[CR25] Girotti F, Carella F, Scigliano G, Grassi MP, Soliveri P, Giovannini P, Parati E, Caraceni T (1984). Effect of neuroleptic treatment on involuntary movements and motor performances in Huntington's disease. J Neurol Neurosurg Psychiatry.

[CR26] Glick D, Barth S, Macleod KF (2010). Autophagy: cellular and molecular mechanism. J Pathol.

[CR27] Gloerich M, Bos JL (2010). Epac: defining a new mechanism for cAMP action. Annu Rev Pharmacol Toxicol.

[CR28] Goll DE, Thompson VF, Li H, Wei W, Cong J (2003). The calpain system. Physiol Rev.

[CR29] Gordon PB, Holen I, Fosse M, Røtnes JS, Seglen PO (1993). Dependence of hepatocytic autophagy on intracellularly sequestered calcium. J Biol Chem.

[CR30] Guo YJ, Dong SY, Cui XX, Feng Y, Liu T, Yin M, Kuo SH, Tan EK, Zhao WJ, Wu YC (2016). Resveratrol alleviates MPTP-induced motor impairments and pathological changes by autophagic degradation of α-synuclein via SIRT1-deacetylated LC3. Mol Nutr Food Res.

[CR31] Gutierrez MG, Munafó DB, Berón W, Colombo MI (2004). Rab7 is required for the normal progression of the autophagic pathway in mammalian cells. J Cell Sci.

[CR32] Hara T, Takamura A, Kishi C, Iemura S, Natsume T, Guan JL, Mizushima N (2008). FIP200, a ULK1 interacting protein, is required for autophagosome formation in mammalian cells. J Cell Biol.

[CR33] Hardie DG (2007). AMP-activated/SNF1 protein kinases: conserved guardians of cellular energy. Nat Rev Mol Cell Biol.

[CR34] Hayashi-Nishino M, Fujita N, Noda T, Yamaguchi A, Yoshimori T, Yamamoto A (2009). A subdomain of the endoplasmic reticulum forms a cradle for autophagosome formation. Nat Cell Biol.

[CR35] Hill MP, Brotchie JM (1999). The adrenergic receptor agonist, clonidine, potentiates the anti-parkinsonian action of the selective κ-opioid receptor agonist, enadoline, in the monoamine-depleted rat. Br J Pharmacol.

[CR36] Ho DJ, Calingasan NY, Wille E, Dumont M, Beal MF (2010). Resveratrol protects against peripheral deficits in a mouse model of Huntington's disease. Exp Neurol.

[CR37] Höllerhage M, Goebel JN, de Andrade A, Hildebrandt T, Dolga A, Culmsee C, Oertel WH, Hengerer B, Höglinger GU (2014). Trifluoperazine rescues human dopaminergic cells from wild-type α-synuclein-induced toxicity. Neurobiol Aging.

[CR38] Horvath J, Coeytaux A, Jallon P, Landis T, Temperli P, Burkhard PR (2005). Carbamazepine encephalopathy masquerading as Creutzfeldt-Jakob disease. Neurology.

[CR39] Hosokawa N, Sasaki T, Iemura SI, Natsume T, Hara T, Mizushima N (2009). Atg101, a novel mammalian autophagy protein interacting with Atg13. Autophagy.

[CR40] Hosokawa N, Hara T, Kaizuka T, Kishi C, Takamura A, Miura Y, Iemura S, Natsume T, Takehana K, Yamada N, Guan JL, Oshiro N, Mizushima N (2009). Nutrient-dependent mTORC1 association with the ULK1–Atg13–FIP200 complex required for autophagy. Mol Biol Cell.

[CR41] Høyer-Hansen M, Jäättelä M (2007). AMP-activated protein kinase: a universal regulator of autophagy?. Autophagy.

[CR42] Huang J, Manning BD (2008). The TSC1-TSC2 complex: a molecular switchboard controlling cell growth. Biochem J.

[CR43] Inoki K, Zhu T, Guan KL (2003). TSC2 mediates cellular energy response to control cell growth and survival. Cell.

[CR44] Inoki K, Ouyang H, Zhu T, Lindvall C, Wang Y, Zhang X, Yang Q, Bennett C, Harada Y, Stankunas K (2006). TSC2 integrates Wnt and energy signals via a coordinated phosphorylation by AMPK and GSK3 to regulate cell growth. Cell.

[CR45] Jacinto E, Loewith R, Schmidt A, Lin S, Rüegg MA, Hall A, Hall MN (2004). Mammalian TOR complex 2 controls the actin cytoskeleton and is rapamycin insensitive. Nat Cell Biol.

[CR46] Jung CH, Ro SH, Chao J, Otto NM, Kim DH (2010). mTOR regulation of autophagy. FEBS Lett.

[CR47] Kalonia H, Kumar P, Kumar A (2011). Attenuation of proinflammatory cytokines and apoptotic process by verapamil and diltiazem against quinolinic acid induced Huntington like alterations in rats. Brain Res.

[CR48] Kaushik S, Bandyopadhyay U, Sridhar S, Kiffin R, Martinez-Vicente M, Kon M, Orenstein SJ, Wong E, Cuervo AM (2011). Chaperon-mediated autophagy at a glance. J Cell Sci.

[CR49] Kawata Y, Okada M, Murakami T, Kamata A, Zhu G, Kaneko S (2001). Pharmacological discrimination between effects of carbamazepine on hippocampal basal, Ca2+- and K+-evoked serotonin release. Br J Pharmacol.

[CR50] Kim R (2005). Recent advances in understanding the cell death pathways activated by anticancer therapy. Cancer.

[CR51] Kim J, Kim E (2016). Rag GTPase in amino acid signaling. Amino Acids.

[CR52] Kim DH, Sarbassov DD, Ali SM, King JE, Latek RR, Erdjument-Bromage H, Tempst P, Sabatini DM (2002). mTOR interacts with raptor to form a nutrient-sensitive complex that signals to the cell growth machinery. Cell.

[CR53] Kim KH, Dodsworth C, Paras A, Burton BK (2013). High dose genistein aglycone therapy is safe in patients with mucopolysaccharidoses involving the central nervous system. Mol Genet Metab.

[CR54] Kim YD, Jeong EI, Nah J, Yoo SM, Lee WJ, Kim Y, Moon S, Hong SH, Jung YK (2017). Pimozide reduces toxic forms of tau in TauC3 mice via 5' adenosine monophosphate-activated protein kinase-mediated autophagy. J Neurochem.

[CR55] Kirisako T, Ichimura Y, Okada H, Kabeya Y, Mizushima N, Yoshimori T, Ohsumi M, Takao T, Noda T, Ohsumi Y (2005). The reversible modification regulates the membrane-binding state of Apg8/Aut7 essential for autophagy and the cytoplasm to vacuole targeting pathway. J Cell Biol.

[CR56] Klionsky DJ (2005). The molecular machinery of autophagy: unanswered questions. J Cell Sci.

[CR57] Köchl R, Hu XW, Chan EY, Tooze SA (2006). Microtubules facilitate autophagosome formation and fusion of autophagosomes with endosomes. Traffic.

[CR58] Kondo Y, Kanzawa T, Sawaya R, Kondo S (2005). The role of autophagy in cancer development and response to therapy. Nat Rev Cancer.

[CR59] Kost A, Kasprowska D, Łabuzek K, Wiaderkiewicz R, Gabryel B (2011). Autofagia w niedokrwieniu mózgu. Postepy Hig Med Dosw.

[CR60] Krüger U, Wang Y, Kumar S, Mandelkow EM (2012). Autophagic degradation of tau in primary neurons and its enhancement by trehalose. Neurobiol Aging.

[CR61] Kuma A, Hatano M, Matsui M, Yamamoto A, Nakaya H, Yoshimori T, Ohsumi Y, Tokuhisa T, Mizushima N (2004). The role of autophagy during the early neonatal starvation period. Nature.

[CR62] Kumar P, Padi SS, Naidu PS, Kumar A (2006). Effect of resveratrol on 3-nitropropionic acid-induced biochemical and behavioural changes: possible neuroprotective mechanisms. Behav Pharmacol.

[CR63] Lan DM, Liu FT, Zhao J, Chen Y, Wu JJ, Ding ZT, Yue ZY, Ren HM, Jiang YP, Wang J (2012). Effect of trehalose on PC12 cells overexpressing wild-type or A53T mutant α-synuclein. Neurochem Res.

[CR64] Lauterbach EC (2013). Neuroprotective effects of psychotropic drugs in Huntington's disease. Int J Mol Sci.

[CR65] Legendre C, Casagrande F, Andrieu T, Dormont D, Clayette P (2007). Sodium valproate does not augment Prpsc in murine neuroblastoma cells. Neurotox Res.

[CR66] Lermontova NN, Lukoyanov NV, Serkova TP, Lukoyanova EA, Bachurin SO (2000). Dimebon improves learning in animals with experimental Alzheimer's disease. Bull Exp Biol Med.

[CR67] Lermontova NN, Redkozubov AE, Shevtsova EF, Serkova TP, Kireeva EG, Bachurin SO (2001). Dimebon and tacrine inhibit neurotoxic action of beta-amyloid in culture and block L-type Ca(2+) channels. Bull Exp Biol Med.

[CR68] Li XZ, Chen XP, Zhao K, Bai LM, Zhang H, Zhou XP (2013). Therapeutic effects of valproate combined with lithium carbonate on MPTP-induced parkinsonism in mice: possible mediation through enhanced autophagy. Int J Neurosci.

[CR69] Liang C, Jung JU (2010). Autophagy genes as tumor suppressors. Curr Opin Cell Biol.

[CR70] Linares GR, Chiu CT, Scheuing L, Leng Y, Liao HM, Maric D, Chuang DM (2016). Preconditioning mesenchymal stem cells with the mood stabilizers lithium and valproic acid enhances therapeutic efficacy in a mouse model of Huntington's disease. Exp Neurol.

[CR71] Liu XW, Su Y, Zhu H, Cao J, Ding WJ, Zhao YC, He QJ, Yang B (2010). HIF-1a-dependent autophagy protects HeLa cells from fenretinide (4-HPR)-induced apoptosis in hypoxia. Pharmacol Res.

[CR72] Long Z, Zeng Q, Wang K, Sharma A, He G (2016). Gender difference in valproic acid-induced neuroprotective effects on APP/PS1 double transgenic mice modeling Alzheimer's disease. Acta Biochim Biophys Sin Shanghai.

[CR73] Majerus PW (1992). Inositol phosphate biochemistry. Annu Rev Biochem.

[CR74] Majumder S, Richardson A, Strong R, Oddo S (2011). Inducing autophagy by rapamycin before, but not after, the formation of plaques and tangles ameliorates cognitive deficits. PLoS One.

[CR75] Malinowska M, Wilkinson FL, Bennett W, Langford-Smith KJ, O'Leary HA, Jakobkiewicz-Banecka J, Wynn R, Wraith JE, Wegrzyn G, Bigger BW (2009). Genistein reduces lysosomal storage in peripheral tissues of mucopolysaccharide IIIB mice. Mol Genet Metab.

[CR76] Malinowska M, Wilkinson FL, Langford-Smith KJ, Langford-Smith A, Brown JR, Crawford BE, Vanier MT, Grynkiewicz G, Wynn RF, Wraith JE, Wegrzyn G, Bigger BW (2010). Genistein improves neuropathology and corrects behaviour in a mouse model of neurodegenerative metabolic disease. PLoS One.

[CR77] Mariňo G, López-Otín C (2004). Autophagy: molecular mechanism, physiological functions and relevance in human pathology. Cell Mol Life Sci.

[CR78] Massacesi C, di Tomaso E, Fretault N, Hirawat S (2013). Challenges in the clinical development of PI3K inhibitors. Ann N Y Acad Sci.

[CR79] Mehrpour M, Esclatine A, Beau I, Codogno P (2010). Overview of macroautophagy regulation in mammalian cells. Cell Res.

[CR80] Meijer AJ, Codogno P (2004). Regulation and role of autophagy in mammalian cells. Int J Biochem Cell Biol.

[CR81] Meijer AJ, Codogno P (2007). AMP-activated protein kinase and autophagy. Autophagy.

[CR82] Meléndez A, Tallóczy Z, Seaman M, Eskelinen EL, Hall DH, Levine B (2003). Autophagy genes are essential for dauer development and life-span extension in C. elegans. Science.

[CR83] Menzies FM, Garcia-Arencibia M, Imarisio S, O'Sullivan NC, Ricketts T, Kent BA, Rao MV, Lam W, Green-Thompson ZW, Nixon RA, Saksida LM, Bussey TJ, O'Kane CJ, Rubinsztein DC (2015). Calpain inhibition mediates autophagy-dependent protection against polyglutamine toxicity. Cell Death Differ.

[CR84] Mercer CA, Kaliappan A, Dennis PB (2009). A novel, human Atg13 binding protein, Atg101, interacts with ULK1 and is essential for macroautophagy. Autophagy.

[CR85] Mijaljica D, Prescott M, Devenish RJ (2011). Microautophagy in mammalian cells: revisiting a 40-year-old conundrum. Autophagy.

[CR86] Miller RG, Shepherd R, Dao H, Khramstov A, Mendoza M, Graves J, Smith S (1996). Controlled trial of nimodipine in amyotrophic lateral sclerosis. Neuromuscul Disord.

[CR87] Miller RG, Smith SA, Murphy JR, Brinkmann JR, Graves J, Mendoza M, Sands ML, Ringel SP (1996). A clinical trial of verapamil in amyotrophic lateral sclerosis. Muscle Nerve.

[CR88] Milutinović A (2016). Lithium chloride could aggravate brain injury caused by 3-nitropropionic acid. Bosn J Basic Med Sci.

[CR89] Mitterreiter S, Page RM, Kamp F, Hopson J, Winkler E, Ha HR, Hamid R, Herms J, Mayer TU, Nelson DJ, Steiner H, Stahl T, Zeitschel U, Rossner S, Haass C, Lichtenthaler SF (2010). Bepridil and amiodarone simultaneously target the Alzheimer's disease beta- and gamma-secretase via distinct mechanisms. J Neurosci.

[CR90] Mothi M, Sampson S (2013). Pimozide for schizophrenia or related psychoses. Cochrane Database Syst Rev.

[CR91] Murphy R, Freedman JE (2001). Morphine and clonidine activate different K+ channels on rat amygdala neurons. Eur J Pharmacol.

[CR92] Nakatogawa H, Suzuki K, Kamada Y, Ohsumi Y (2009). Dynamics and diversity in autophagy mechanism: lessons from yeast. Nat Rev Mol Cell Biol.

[CR93] Nicklin P, Bergman P, Zhang B, Triantafellow E, Wang H, Nyfeler B, Yang H, Hild M, Kung C, Wilson C (2009). Bidirectional transport of amino acids regulates mTOR and autophagy. Cell.

[CR94] Nimmrich V, Eckert A (2013). Calcium channel blockers and dementia. Br J Pharmacol.

[CR95] Ozcelik S, Fraser G, Castets P, Schaeffer V, Skachokova Z, Breu K, Clavaguera F, Sinnreich M, Kappos L, Goedert M, Tolnay M, Winkler DT (2013). Rapamycin attenuates the progression of tau pathology in P301S tau transgenic mice. PLoS One.

[CR96] Paris D, Bachmeier C, Patel N, Quadros A, Volmar CH, Laporte V, Ganey J, Beaulieu-Abdelahad D, Ait-Ghezala G, Crawford F, Mullan MJ (2011). Selective antihypertensive dihydropyridines lower Aβ accumulation by targeting both the production and the clearance of Aβ across the blood-brain barrier. Mol Med.

[CR97] Pattingre S, Tassa A, Qu X, Garuti R, Liang XH, Mizushima N, Packer M, Schneider MD, Levine B (2005). Bcl-2 antiapoptotic proteins inhibit Beclin 1-dependent autophagy. Cell.

[CR98] Pattingre S, Espert L, Biard-Piechaczyk M, Codogno P (2008). Regulation of macroautophagy by mTOR and Beclin 1 complexes. Biochimie.

[CR99] Perera ND, Sheean RK, Lau CL, Shin YS, Beart PM, Horne MK, Turner BJ (2017). Rilmenidine promotes MTOR-independent autophagy in the mutant SOD1 mouse model of amyotrophic lateral sclerosis without slowing disease progression. Autophagy.

[CR100] Perucho J, Gómez A, Muñoz MP, de Yébenes JG, Mena MÁ, Casarejos MJ (2016). Trehalose rescues glial cell dysfunction in striatal cultures from HD R6/1 mice at early postnatal development. Mol Cell Neurosci.

[CR101] Pierzynowska K, Gaffke L, Hać A, Mantej J, Niedziałek N, Brokowska J, Węgrzyn G (2018) Correction of Huntington's disease phenotype by genistein-induced autophagy in the cellular model. NeuroMolecular Med 20:112-123. 10.1007/s12017-018-8482-1 10.1007/s12017-018-8482-1 PMC5834590 29435951

[CR102] Piotrowska E, Jakobkiewicz-Banecka J, Baranska S, Tylki-Szymanska A, Czartoryska B, Wegrzyn A, Wegrzyn G (2006). Genistein-mediated inhibition of glycosaminoglycan synthesis as a basis for gene expression-targeted isoflavone therapy for mucopolysaccharidoses. Eur J Hum Genet.

[CR103] Porras G, Li Q, Bezard E (2012). Modeling Parkinson’s Disease in Primates: The MPTP Model. Cold Spring Harb Perspect Med.

[CR104] Qu X, Zou Z, Sun Q, Luby-Phelps K, Cheng P, Hogan RN, Gilpin C, Levine B (2007). Autophagy gene-dependent clearance of apoptotic cells during embryonic development. Cell.

[CR105] Rao MV, Mohan PS, Peterhoff CM, Yang DS, Schmidt SD, Stavrides PH, Campbell J, Chen Y, Jiang Y, Paskevich PA, Cataldo AM, Haroutunian V, Nixon RA (2008). Marked calpastatin (CAST) depletion in Alzheimer's disease accelerates cytoskeleton disruption and neurodegeneration: neuroprotection by CAST overexpression. J Neurosci.

[CR106] Ravikumar B, Duden R, Rubinsztein DC (2002). Aggregate-prone proteins with polyglutamine and polyalanine expansions are degraded by autophagy. Hum Mol Genet.

[CR107] Ravikumar B, Vacher C, Berger Z, Davies JE, Luo S, Oroz LG, Scaravilli F, Easton DF, Duden R, O'Kane CJ, Rubinsztein DC (2004). Inhibition of mTOR induces autophagy and reduces toxicity of polyglutamine expansions in fly and mouse models of Huntington disease. Nat Genet.

[CR108] Ravikumar B, Sarkar S, Davies JE, Futter M, Garcia-Arencibia M, Green-Thompson ZW (2010). Regulation of mammalian autophagy in physiology and pathophysiology. Physiol Rev.

[CR109] Reggiori F, Klionsky D (2005). Autophagosomes: biogenesis from scratch?. Cell Biology.

[CR110] Renna M, Jimenez-Sanchez M, Sarkar S, Rubinsztein DC (2010). Chemical inducers of autophagy that enhance the clearance of mutant proteins in neurodegenerative diseases. J Biol Chem.

[CR111] Renvoize EB, Kent J, Klar HM (1987). Delusional infestation and dementia: a case report. Br J Psychiatry.

[CR112] Ricci MS, Zong WX (2006). Chemotherapeutic Approaches for Targeting Cell Death Pathways. Oncologist.

[CR113] Roczniak-Ferguson A, Petit CS, Froehlich F, Qian S, Ky J, Angarola B, Walther TC, Ferguson SM (2012). The transcription factor TFEB links mTORC1 signaling to transcriptional control of lysosome homeostasis. Sci Signal.

[CR114] Rodríguez-Navarro JA, Rodríguez L, Casarejos MJ, Solano RM, Gómez A, Perucho J, Cuervo AM, García de Yébenes J, Mena MA (2010). Trehalose ameliorates dopaminergic and tau pathology in parkin deleted/tau overexpressing mice through autophagy activation. Neurobiol Dis.

[CR115] Rose C, Menzies FM, Renna M, Acevedo-Arozena A, Corrochano S, Sadiq O, Brown SD, Rubinsztein DC (2010). Rilmenidine attenuates toxicity of polyglutamine expansions in a mouse model of Huntington's disease. Hum Mol Genet.

[CR116] Roy S, Debnath J (2010). Autophagy and Tumorigenesis. Semin Immunopathol.

[CR117] Sakai Y, Koller A, Rangell LK, Keller GA, Subramani S (1998). Peroxisome degradation by microautophagy in Pichia pastoris: identification of specific steps and morphological intermediates. J Cell Biol.

[CR118] Sancak Y, Peterson TR, Shaul YD, Lindquist RA, Thoreen CC, Bar-Peled L, Sabatini DM (2008). The Rag GTPases bind raptor and mediate amino acid signaling to mTORC1. Science.

[CR119] Sarbassov DD, Ali SM, Sabatini DM (2005). Growing roles for the mTOR pathway. Curr Opin Cell Biol.

[CR120] Sarkar S (2013). Regulation of autophagy by mTOR-dependent and mTOR-independent pathways: autophagy dysfunction in neurodegenerative diseases and therapeutic application of autophagy enhancers. Biochem Soc Trans.

[CR121] Sarkar S, Rubinsztein DC (2008). Huntington's disease: degradation of mutant huntingtin by autophagy. FEBS J.

[CR122] Sarkar S, Floto RA, Berger Z, Imarisio S, Cordenier A, Pasco M, Cook LJ, Rubinsztein DC (2005). Lithium induces autophagy by inhibiting inositol monophosphatase. J Cell Biol.

[CR123] Sarkar S, Krishna G, Imarisio S, Saiki S, O'Kane CJ, Rubinsztein DC (2008). A rational mechanism for combination treatment of Huntington's disease using lithium and rapamycin. Hum Mol Genet.

[CR124] Sarkar S, Korolchuk VI, Renna M, Imarisio S, Fleming A, Williams A, Garcia-Arencibia M, Rose C, Luo S, Underwood BR, Kroemer G, O'Kane CJ, Rubinsztein DC (2011). Complex inhibitory effects of nitric oxide on autophagy. Mol Cell.

[CR125] Sato-Kusubata K, Yajima Y, Kawashima S (2000). Persistent activation of Gsa through limited proteolysis by calpain. Biochem J.

[CR126] Schaeffer V, Lavenir I, Ozcelik S, Tolnay M, Winkler DT, Goedert M (2012). Stimulation of autophagy reduces neurodegeneration in a mouse model of human tauopathy. Brain.

[CR127] Scheuing L, Chiu CT, Liao HM, Linares GR, Chuang DM (2014). Preclinical and clinical investigations of mood stabilizers for Huntington's disease: what have we learned?. Int J Biol Sci.

[CR128] Schiebler M, Brown K, Hegyi K, Newton SM, Renna M, Hepburn L, Klapholz C (2015). Functional drug screening reveals anticonvulsants as enhancers of mTOR-independent autophagic killing of Mycobacterium tuberculosis through inositol depletion. EMBO Mol Med.

[CR129] Schneider JS, Tinker JP, Decamp E (2010). Clondine improves attentional and memory components of delay response performance in a model of early parkinsonism. Behav Brain Res.

[CR130] Schoch KM, von Reyn CR, Bian J, Telling GC, Meaney DF, Saatman KE (2013). Brain injury-induced proteolysis is reduced in a novel calpastatin-overexpressing transgenic mouse. J Neurochem.

[CR131] Seo J, Jo SA, Hwang S, Byun CJ, Lee HJ, Cho DH, Kim D, Koh YH, Jo I (2013). Trichostatin A epigenetically increases calpastatin expression and inhibits calpain activity and calcium-induced SH-SY5Y neuronal cell toxicity. FEBS J.

[CR132] Shaked GM, Engelstein R, Avraham I, Rosenmann H, Gabizon R (2002). Valproic acid treatment results in increased accumulation of prion proteins. Ann Neurol.

[CR133] Shaltiel G, Shamir A, Shapiro J, Ding D, Dalton E, Bialer M, Harwood AJ, Belmaker RH, Greenberg ML, Agam G (2004). Valproate decreases inositol biosynthesis. Biol Psychiatry.

[CR134] Shariatpanahi M, Khodagholi F, Ashabi G, Aghazadeh Khasraghi A, Azimi L, Abdollahi M, Ghahremani MH, Ostad SN, Noorbakhsh F, Sharifzadeh M (2015). Ameliorating of memory impairment and apoptosis in amyloid β-injected rats via inhibition of nitric oxide synthase: possible participation of autophagy. Iran J Pharm Res.

[CR135] Shaw RJ, Bardeesy N, Manning BD, Lopez L, Kosmatka M, DePinho RA, Cantley LC (2004). The LKB1 tumor suppressor negatively regulates mTOR signaling. Cancer Cell.

[CR136] Shen D, Coleman J, Chan E, Nicholson TP, Dai L, Sheppard PW, Patton WF (2011). Novel cell- and tissue-based assays for detecting misfolded and aggregated protein accumulation within aggresomes and inclusion bodies. Cell Biochem Biophys.

[CR137] Shimada K, Motoi Y, Ishiguro K, Kambe T, Matsumoto SE, Itaya M, Kunichika M, Mori H, Shinohara A, Chiba M, Mizuno Y, Ueno T, Hattori N (2012). Long-term oral lithium treatment attenuates motor disturbance in tauopathy model mice: implications of autophagy promotion. Neurobiol Dis.

[CR138] Shintani T, Klionsky DJ (2004). Autophagy in health and disease: a double-edged sword. Science.

[CR139] Sirdharan S, Jain K, Basu A (2011). Regulation autophagy by kinases. Cancers (Basel).

[CR140] Sorial ME, El Sayed NSED (2017). Protective effect of valproic acid in streptozotocin-induced sporadic Alzheimer's disease mouse model: possible involvement of the cholinergic system. Naunyn Schmiedeberg's Arch Pharmacol.

[CR141] Spilman P, Podlutskaya N, Hart MJ, Debnath J, Gorostiza O, Bredesen D, Richardson A, Strong R, Galvan V (2010). Inhibition of mTOR by rapamycin abolishes cognitive deficits and reduces amyloid-beta levels in a mouse model of Alzheimer's disease. PLoS One.

[CR142] Stitt TN, Drujan D, Clarke BA, Panaro F, Timofeyva Y, Kline WO, Gonzalez M, Yancopoulos GD, Glass DJ (2004). The IGF-1/PI3K/Akt pathway prevents expression of muscle atrophy-induced ubiquitin ligases by inhibiting FOXO transcription factors. Mol Cell.

[CR143] Stokes HB (1975). Trifluoroperazine for the symptomatic treatment of chorea. Dis Nerv Syst.

[CR144] Sweetlove M (2012). Phase III CONCERT Trial of Latrepirdine. Pharmaceutical Medicine.

[CR145] Tanaka Y, Guhde G, Suter A, Eskelinen EL, Hartmann D, Lüllmann-Rauch R, Janssen PM, Blanz J, von Figura K, Saftig P (2000) Accumulation of autophagic vacuoles and cardiomyopathy in LAMP-2-deficient mice. Nature 406:902–906. 10.1038/35022595 10.1038/35022595 10972293

[CR146] Tanida I (2011). Autophagy basics. Microbiol Immunol.

[CR147] Tanida I, Minematsu-Ikeguchi N, Ueno T, Kominami E (2005). Lysosomal turnover, but not a cellular level of endogenous LC3 is a marker for autophagy. Autophagy.

[CR148] Tian Y, Bustos V, Flajolet M, Greengard P (2011). A small-molecule enhancer of autophagy decreases levels of Abeta and APP-CTF via Atg5-dependent autophagy pathway. FASEB J.

[CR149] Tribouillard-Tanvier D, Béringue V, Desban N, Gug F, Bach S, Voisset C, Galons H, Laude H, Vilette D, Blondel M (2008). Antihypertensive drug guanabenz is active in vivo against both yeast and mammalian prions. PLoS One.

[CR150] Tsukamoto S, Kuma A, Murakami M, Kishi C, Yamamoto A, Mizushima N (2008). Autophagy is essential for preimplantation development of mouse embryos. Science.

[CR151] Túnez I, Tasset I, Pérez-De La Cruz V, Santamaría A (2010). 3-Nitropropionic acid as a tool to study the mechanisms involved in Huntington's disease: past, present and future. Molecules.

[CR152] Underwood B, Green-Thompson ZW, Pugh PJ, Lazic SE, Mason SL, Griffin J, Jones PS, Rowe JB, Rubinsztein DC, Barker RA (2017). An open-label study to assess the feasibility and tolerability of rilmenidine for the treatment of Huntington's disease. J Neurol.

[CR153] Vellai T (2009). Autophagy genes and ageing. Cell Death Differ.

[CR154] Vidal RL, Matus S, Bargsted L, Hetz C (2014). Targeting autophagy in neurodegenerative diseases. Trends Pharmacol Sci.

[CR155] Vidoni C, Secomandi E, Castiglioni A, Melone MAB, Isidoro C (2017). Resveratrol protects neuronal-like cells expressing mutant Huntingtin from dopamine toxicity by rescuing ATG4-mediated autophagosome formation. Neurochem Int pii.

[CR156] Vodicka P, Chase K, Iuliano M, Tousley A, Valentine DT, Sapp E, Kegel-Gleason KB, Sena-Esteves M, Aronin N, DiFiglia M (2016). Autophagy Activation by Transcription Factor EB (TFEB) in Striatum of HDQ175/Q7 Mice. J Huntingtons Dis.

[CR157] Webb JL, Ravikumar B, Atkins J, Skepper JN, Rubinsztein DC (2003). Alpha-Synuclein is degraded by both autophagy and the proteasome. J Biol Chem.

[CR158] Wei Y, Pattingre S, Sinha S, Bassik M, Levine B (2008). JNK1-mediated phosphorylation of Bcl-2 regulates starvation-induced autophagy. Mol Cell.

[CR159] Williams RS, Bate C (2016). An in vitro model for synaptic loss in neurodegenerative diseases suggests a neuroprotective role for valproic acid via inhibition of cPLA2 dependent signalling. Neuropharmacology.

[CR160] Williams RSB, Bate C (2018). Valproic acid and its congener propylisopropylacetic acid reduced the amount of soluble amyloid-β oligomers released from 7PA2 cells. Neuropharmacology.

[CR161] Williams RS, Cheng L, Mudge AW, Harwood AJ (2002). A common mechanism of action for three mood-stabilizing drugs. Nature.

[CR162] Williams A, Sarkar S, Cuddon P, Ttofi EK, Saiki S, Siddiqi FH, Jahreiss L, Fleming A, Pask D, Goldsmith P, O'Kane CJ, Floto RA, Rubinsztein DC (2008). Novel targets for Huntington's disease in an mTOR-independent autophagy pathway. Nat Chem Biol.

[CR163] Wong ASL, Cheung ZH, Ip NY (2011). Molecular machinery of macroautophagy and its deregulation in diseases. Biochim Biophys Acta.

[CR164] Wu S, Zheng SD, Huang HL, Yan LC, Yin XF, Xu HN, Zhang KJ, Gui JH, Chu L, Liu XY (2013). Lithium down-regulates histone deacetylase 1 (HDAC1) and induces degradation of mutant huntingtin. J Biol Chem.

[CR165] Yang Z, Klionsky DJ (2010). Mammalian autophagy: core molecular machinery and signaling regulation. Curr Opin Cell Biol.

[CR166] Yang Y, Liang ZQ, Gu ZL, Qin ZH (2005). Molecular mechanism and regulation of autophagy. Acta Pharmacol Sin.

[CR167] Yang W, Lu J, Weng J, Jia W, Ji L, Xiao J, Shan Z, Liu J, Tian H, Ji Q, Zhu D, Ge J, Lin L, Chen L, Guo X, Zhao Z, Li Q, Zhou Z, Shan G, He J (2010). Prevalence of Diabetes among Men and Women in China. N Engl J Med.

[CR168] Yorimitsu T, Klionsky DJ (2005). Autophagy: molecular machinery for self-eating. Cell Death Differ.

[CR169] Zarzynska J, Motyl T (2008). Apoptosis and Autophagy in Involuting Bovine Mammary Gland. J Physiol Pharmacol.

[CR170] Zhang L, Yu J, Pan H, Hu P, Hao Y, Cai W, Zhu H, Yu AD, Xie X, Ma D, Yuan J (2007). Small molecule regulators of autophagy identified by an image-based high-throughput screen. Proc Natl Acad Sci U S A.

[CR171] Zhang X, Heng X, Li T, Li L, Yang D, Zhang X, Du Y, Doody RS, Le W (2011). Long-term treatment with lithium alleviates memory deficits and reduces amyloid-β production in an aged Alzheimer's disease transgenic mouse model. J Alzheimers Dis.

[CR172] Zhang X, Li L, Chen S, Yang D, Wang Y, Zhang X, Wang Z, Le W (2011). Rapamycin treatment augments motor neuron degeneration in SOD1(G93A) mouse model of amyotrophic lateral sclerosis. Autophagy.

[CR173] Zhang L, Wang L, Wang R, Gao Y, Che H, Pan Y, Fu P (2017). Evaluating the Effectiveness of GTM-1, Rapamycin, and Carbamazepine on Autophagy and Alzheimer Disease. Med Sci Monit.

[CR174] Zhang Y, Nguyen DT, Olzomer EM, Poon GP, Cole NJ, Puvanendran A, Phillips BR, Hesselson D (2017). Rescue of Pink1 Deficiency by Stress-Dependent Activation of Autophagy. Cell Chem Biol.

[CR175] Zhang Y, Wu JY, Weng LH, Li XX, Yu LJ, Xu Y (2017). Valproic acid protects against MPP+-mediated neurotoxicity in SH-SY5Y Cells through autophagy. Neurosci Lett.

[CR176] Zhao J, Zhi X, Pan L, Zhou P (2017) Trehalose Inhibits A53T Mutant α-Synuclein Overexpression and Neurotoxicity in Transduced PC12 Cells. Molecules 22. 10.3390/molecules22081293 10.3390/molecules22081293 PMC6152154 28786917

[CR177] Zheng Q, Huang T, Zhang L, Zhou Y, Luo H, Xu H, Wang X (2016). Dysregulation of Ubiquitin-Proteasome System in Neurodegenerative Diseases. Front Aging Neurosci.

[CR178] Zhong Y, Wang QJ, Li X, Yan Y, Backer JM, Chait BT, Heintz N, Yue Z (2009). Distinct regulation of autophagic activity by Atg 14L and Rubicon 107 associated with Beclin 1-phosphatidylinositol-3-kinase complex. Nat Cell Biol.

[CR179] Zhu L, Li L, Zhang Q, Yang X, Zou Z, Hao B, Marincola FM, Liu Z, Zhong Z, Wang M, Li X, Wang Q, Li K, Gao W, Yao K, Liu Q (2017). NOS1 S-nitrosylates PTEN and inhibits autophagy in nasopharyngeal carcinoma cells. Cell Death Dis.

[CR180] Zoncu R, Bar-Peled L, Efeyan A, Wang S, Sancak Y, Sabatini DM (2011). mTORC1 senses lysosomal amino acids through an inside-out mechanism that requires the vacuolar H+ -ATPase. Science.

